# Multi-Bioinformatics Approach Reveals COX5B-Mediated Modulation of Oxidative Phosphorylation and ROS Levels by Water and Ethanol Extracts of Schisandrae Fructus in SW1783 Cells

**DOI:** 10.3390/antiox15060728

**Published:** 2026-06-09

**Authors:** Musun Park, No Soo Kim, Jin-Mu Yi, Seongwon Cha

**Affiliations:** 1 Korean Medicine Data Division, Korea Institute of Oriental Medicine, 1672 Yuseong-daero, Yuseong-gu, Daejeon 34054, Republic of Korea; scha@kiom.re.kr; 2 NeuroGlymph Imaging and Modulation Center, Korea Institute of Oriental Medicine, 1672 Yuseong-daero, Yuseong-gu, Daejeon 34054, Republic of Korea; nosookim@kiom.re.kr; 3 Korean Medicine Convergence Research Division, Korea Institute of Oriental Medicine, 1672 Yuseong-daero, Yuseong-gu, Daejeon 34054, Republic of Korea; jmyi@kiom.re.kr

**Keywords:** Schisandrae Fructus, oxidative phosphorylation, transcriptome, multi-bioinformatics analyses, COX5B

## Abstract

Schisandrae Fructus is a traditional medicinal herb used to suppress hyperactivity and exhibits a wide range of therapeutic effects and few side effects; however, the molecular mechanism of action engaged by the multiple compounds found within Schisandrae Fructus remains insufficiently studied. Herein, we aimed to confirm the inhibition of oxidative phosphorylation by hot water and ethanol extracts of Schisandrae Fructus and determine the activity and pathways affected by each extract. To refine target prediction, centrality analysis was performed using drug–target interactions, protein–protein interactions, and integrated networks. Our analyses revealed that oxidative phosphorylation was inhibited in the water extract, and COX5B was predicted to be a common target. Centrality analysis revealed COX5B’s high score, suggesting its role as an efficient mediator of oxidative phosphorylation regulation. In vitro analysis confirmed that COX5B expression, oxidative phosphorylation activity and reactive oxygen species levels were suppressed by the water extract. The water and ethanol extracts of Schisandrae Fructus differentially affected oxidative phosphorylation activity. COX5B, a key target regulated by the water extract of Schisandrae Fructus, emerged as a potential biomarker. These findings suggest that COX5B-mediated oxidative phosphorylation modulation may serve as a key mechanism underlying the anticancer and antiaging effects of Schisandrae Fructus.

## 1. Introduction

Schisandrae Fructus (SF) is the fruit of *Schisandra chinensis* Baillon—a herb used in traditional Asian medicine (TAM) in East Asia. It is mainly prescribed as a medicine for hyperactivity symptoms, such as sweating, thirst, and exhaustion, that can occur during excessive exercise, high solar irradiance, or dehydration [1]. Although SF has traditionally been used for various conditions, recent studies have provided scientific evidence supporting its biological activities and therapeutic potential. Accordingly, SF has been reported to possess antioxidant, anti-inflammatory, neuroprotective, hepatoprotective, skin-protective, anti-diabetic, anti-obesity, anti-aging, and anti-sarcopenia effects [2,3,4,5,6,7,8,9,10,11,12], thus exhibiting a wide range of therapeutic effects and few side effects. Among these biological activities, the antioxidant and neuroprotective effects of SF have attracted particular attention [4]. Oxidative stress and mitochondrial dysfunction are recognized as major mechanisms contributing to neuronal damage and the progression of age-related neurodegenerative disorders [13]. In this context, previous studies have reported that SF shows neuroprotective effects by restoring antioxidant activity and preventing the loss of mitochondrial membrane potential [14]. In addition, oxidative phosphorylation (OXPHOS) is one of the major intracellular sources of reactive oxygen species (ROS), and excessive generation of mitochondrial ROS can induce oxidative damage in neurons and astrocytes, which may contribute to brain damage [15,16,17,18]. Since previous studies have suggested that SF can protect the brain from oxidative damage [19], modulation of OXPHOS-associated oxidative stress in the nervous system may represent one of the potential mechanisms underlying the previously reported neuroprotective effects of SF. Despite increasing evidence supporting the antioxidant and neuroprotective effects of SF, the molecular mechanisms through which multiple compounds in SF regulate OXPHOS activity and OXPHOS-associated oxidative stress remain insufficiently investigated.

In TAM, water has traditionally been used as the main solvent for herbal decoction [20]. However, recent studies have verified the efficacy of ethanol extracts of SF (EESF), in addition to hot water extracts of SF (WESF) [21]. Ethanol is generally preferred in many pharmacological analyses because it can extract hydrophobic compounds that are difficult to extract from hot water [22,23]. However, not many studies have systematically compared the WESF and EESF. In previous studies, the compositions of WESF and EESF were not compared because the two extracts were similar [24]. Another study compared the differences in WESF and EESF composition using liquid chromatography analysis, yet the mechanism of action or specific targets could not be inferred [25]. Therefore, further research is needed to determine the mechanism of action underlying the therapeutic effects of WESF and EESF.

Herbal extracts contain multiple bioactive compounds, making it challenging to identify specific mechanisms of action using conventional methods [26]. Bioinformatics analysis represents a powerful tool for exploring the mechanism of action and targets of mixture compounds [27]. In particular, the presence of multiple active compounds in extracts requires the use of computational polypharmacology (PP) methods based on high-throughput data [28]. Among them, transcriptome analysis facilitates analysis of gene expression changes in drug-treated tissues or cell lines [29,30,31]. Network biology approaches reproduce physiological or pathological models based on networks and predicts mechanisms by utilizing multiple targets predicted for different compounds in the network model [32,33]. In addition, the virtual screening method using large-scale molecular docking can predict direct drug–target interactions, making it particularly useful for PP analysis [34]. Despite the valuable target prediction insights provided by them, bioinformatics analyses can only reproduce limited time points within biological phenomena. For example, docking analysis can only predict a drug–protein interaction, reflecting the initial step of drug signaling, while transcriptome analysis quantifies gene expression changes after treatment, capturing only the end point of the signaling cascade. Therefore, advanced drug mechanism research should include predictive studies using various bioinformatics analysis tools in a complementary manner.

In this study, we aimed to investigate the differences in activities between WESF and EESF using multi-bioinformatics analyses and identify the mechanistic basis of such differences. By comparing transcriptome changes following treatment, we predicted differences in OXPHOS activity between the two extracts. To comprehensively analyze the mechanism of action of WESF, we explored the targets of its strongly hydrophilic components. Through protein–protein interaction (PPI) network analysis, drug–target interaction (DTI) network based on docking analysis, and PP network analysis, we identified the module targeted by strongly hydrophilic compounds and predicted key genes implicated in the mechanism. Finally, through in vitro experiments, we confirmed differences in the expression of key genes regulated by WESF and EESF as well as the changes in OXPHOS activity and ROS levels.

## 2. Materials and Methods

### 2.1. Chemicals and Reagents

Dimethyl sulfoxide (DMSO, D8418), hydrogen peroxide (H_2_O_2_, H1009), oligomycin (O4876), trichloroacetic acid (TCA, T0699), and Triton X-100 (TX-100, T8787) were purchased from Sigma-Aldrich (St. Louis, MO, USA). Pre-made 1 M Tris-HCl buffer, pH7.5 (IBS-BT017-1) and 0.5 M ethylenediaminetetraacetic acid (EDTA, IBS-BE002) were purchased from iNtRON Biotechnology (Seongnam, Republic of Korea). CellROX Green (C10444), fetal bovine serum (FBS, #12483020), Hank’s balanced salt solution (HBSS, #14025-092), penicillin-streptomycin solution (#15140-122), phosphate-buffered saline (PBS, #10010-023), and TrypLE Express (#12604-013) were obtained from Thermo Fisher Scientific (Waltham, MA, USA). Leibovitz’s L-15 basal medium (#30-2008) was purchased from the American Type Culture Collection (ATCC, Manassas, VA, USA). Cell culture flasks and multi-well plates were supplied by Thermo Fisher Scientific (Waltham, MA, USA). QIAzol Lysis Reagent (#79306) was obtained from Qiagen (Germantown, MD, USA).

### 2.2. Preparation of WESF and EESF

Dried SF was supplied by Kwangmyungdang Medicinal Herbs Co. (Ulsan, Republic of Korea) and authenticated through organoleptic evaluation by Dr. Goya Choi, a certified expert designated by the Korea Food and Drug Administration. Species identity was verified using sequence-characterized amplified region (SCAR) markers applied to genomic DNA. A plant voucher specimen (#2-24-0079) was deposited in the Korean Herbarium of Standard Herbal Resources, KIOM (Naju, Republic of Korea). For extraction, the pulverized material was processed by two methods. The WESF was prepared by refluxing at 100 ± 3 °C for 3 h (MS-DM609, Misung Scientific, Yangju, Republic of Korea). The EESF was obtained by maceration in 70% ethanol for 1 h with ultrasonication (VCP-20, Lab Companion, Daejeon, Republic of Korea), repeated twice. Both extracts were filtered through 5 µm cartridge filters (KOC Biotech, Daejeon, Republic of Korea), concentrated at 40 °C using a rotary evaporator (Ev-1020t, SciLab, Seoul, Republic of Korea), and lyophilized (LP20, ilShinBiobase, Dongducheon, Republic of Korea). The extraction yields were 47.4% for WESF and 40.0% for EESF. Dried extracts were homogenized and stored in airtight containers at 4 °C. Voucher specimens of the final extracts were also deposited in the Korean Herbarium of Standard Herbal Resources under registration numbers #3-24-0020 (WESF) and #3-24-0021 (EESF). For in vitro experiments, the extracts were dissolved in PBS containing 2% DMSO in PBS, vortexed for 30 min at 22–24 °C, and sterilized using a 0.22 µm syringe filter. Stock solutions (10 mg/mL) were aliquoted into 1.5 mL tubes and stored at −80 °C until use.

### 2.3. Cell Culture and Drug Treatment for RNA-Sequencing (RNA-Seq)

Human astrocytoma cell line SW1783 (HTB-13) was obtained from ATCC and cultured in a growth medium consisting of Leibovitz’s L-15 basal medium supplemented with 10% heat-inactivated FBS and 100 IU/mL penicillin-100 µg/mL streptomycin at 37 °C without CO_2_ balance. Cells were split every 3–4 days based on confluency. For drug treatment, cells were plated at 1.5 × 10^5^ cells/well in 6-well plates containing 3 mL of growth medium 1 day prior to treatment. WESF was applied at final concentrations of 20 (low), 100 (medium), and 500 µg/mL (high), versus 12.4 (low), 62 (medium), and 310 µg/mL (high) for EESF. The highest doses of WESF and EESF were selected based on preliminary cytotoxicity screening: 500 µg/mL WESF showed no toxicity, while 310 µg/mL EESF corresponded to its IC_20_ value (309.6 µg/mL). PBS with 0.1% DMSO served as the vehicle control. After 24 h of drug treatment, cells were washed thrice with ice-cold PBS. Total RNA was extracted using QIAzol Lysis Reagent, and lysates were stored at −70 °C until RNA-seq.

### 2.4. RNA Preparation for RNA-Seq

The quality of the raw RNA-seq data was assessed using FastQC (v0.11.9) [35]. Adapter sequences were removed from the reads using Trim Galore (v0.6.6) [36]. The cleaned reads were aligned to the human reference genome GRCh38 (hg19) using STAR (v2.7.9.a) [37]. Transcript abundance per gene was quantified using RSEM (v1.3.3) [38] with the gene annotation GRCh38.84. The expected read counts and transcripts per million were used as the gene expression levels for further analyses. Differential gene expression between the treatment and vehicle groups was analyzed using the Wald test implemented in the R package DESeq2 (v1.38.2) [39]. The RNA-seq data were analyzed using R (v4.3.1). RNA sequence data were deposited in the Gene Expression Omnibus under accession number GSE294592.

### 2.5. RNA-Seq Preprocessing Method

Differentially expressed gene (DEG) analysis and Gene set enrichment analysis (GSEA) were performed to compare transcript expression between water and ethanol extracts. First, transcripts with a fold change in expression greater than log_2_(1.5) or less than −log_2_(1.5) and an adjusted *p*-value < 0.05 were selected as DEGs. As the number of DEGs at middle and low doses was extremely small (less than 50), analyses were performed only at the high dose. DEG information extraction was performed using R software (v4.3.1).

GSEA was performed for the high, middle, and low doses of both WESF and EESF. GSEA was performed for curated Kyoto Encyclopedia of Genes and Genomes (KEGG) pathway gene sets in the Molecular Signature Database (MSigDB v7.5.1) using the fgsea package (v1.24) in R with parameters of minimum size 15, maximum size 500, and 100,000 permutations [40,41,42]. The statistical significance of GSEA results was evaluated by adjusting the *p*-value using the Benjamini–Hochberg procedure [43]. The GSEA results were visualized as a heatmap using Pheatmap R package (v1.0.12). Heatmap visualization was performed by selecting only KEGG pathways that had at least two valid values (*p* < 0.05) in six types of extracts (two solvents, three doses). Additional analysis was performed for the OXPHOS pathway, which was derived from the GSEA results with opposite expression patterns between WESF and EESF. That is, only genes belonging to KEGG OXPHOS were selected, and genes whose expression levels decreased under high-dose WESF and increased under high-dose EESF were extracted to confirm expression differences through a heatmap [44]. The Ensemble IDs derived from the analysis results was searched in the GeneCards database [45], and the data that did not properly match the gene symbol by comparing the amino acid sequence were excluded from the analysis (accessed on 21 February 2025). The gene information belonging to OXPHOS used the gene set provided by the “KEGG 2021 Human” dataset on the EnrichR platform [46] (accessed on 18 June 2024). Among the proteins included in the OXPHOS pathway in the “KEGG 2021 Human dataset,” the OXPHOS protein set containing 133 proteins was completed by renaming the gene names that were changed using the Entrez database to the currently used gene symbols [47] (accessed on 8 March 2024). The heatmap of transcriptome expression was visualized using the python seaborn library (v0.13.2).

### 2.6. SF Compound Collection

To collect SF compounds from public databases, we searched TCMSP [48], BATMAN-TCM [49], and TM-MC [50] (accessed on 12 April 2023, respectively). First, among the collected compounds, only verified compounds whose compound CID information was searched in the PubChem database were analyzed. In addition, (1) compounds that cannot be used as drugs because their molecular weight is too large and (2) compounds whose predicted 3D structures are provided in the PubChem database for molecular docking analysis were selected as valid SF compounds [51]. Compound CID information, 3D structures, and 3D sdf file download were performed using the Pubchempy library.

To classify hydrophilicity and hydrophobicity, basic property information was collected to identify the selected valid compounds of SF. Molecular formula, molecular weight, Isomeric SMILES, InChI, InChIKey, IUPAC name, and XLogP information were collected using the Pubchempy library. Among them, XLogP—which indicates the degree of partition coefficient—was used to classify the valid compounds into hydrophilic and hydrophobic [52,53]. The higher the water–octanol partition coefficient, the stronger the hydrophobicity of the compound, and the lower the water–octanol partition coefficient, the stronger the hydrophilicity of the compound. However, a clear standard for the coefficient value that can distinguish between hydrophilicity and hydrophobicity has not yet been presented. Therefore, in this study, the polarity of valid compounds was evaluated using the relative XlogP value. The XLogP values of valid compounds were divided into four quartiles, and the top 25% group with the highest values was defined as strongly hydrophobic. The next 50% group was classified as weakly hydrophobic, the third quartile as weakly hydrophilic, and the last group as strongly hydrophilic. Since there is no universally accepted XLogP cut-off for distinguishing strong versus weak hydrophilicity, applying a fixed threshold may introduce arbitrary classification bias. Specifically, excessively imbalanced group sizes create differences in sampling density across groups [54]. This leads to unequal opportunities for observation and increases the likelihood that the observed interaction patterns reflect group composition rather than relative polarity. Therefore, in this study, quartile-based stratification was adopted to construct groups with balanced numbers of compounds, ensure similar opportunities for observation across groups, and explore relative polarity-dependent patterns.

### 2.7. Large-Scale Molecular Docking

To collect OXPHOS-related proteins, the 3D structures of proteins included in the OXPHOS protein set, which includes 133 proteins, were obtained using the AlphaFold database [55] (alphafold_v2, https://ftp.ebi.ac.uk/pub/databases/alphafold/v2/, accessed on 25 March 2024). The 3D pdb structure of the protein corresponding to the human-derived protein (Reference proteome ID: UP000005640) searched in the AlphaFold database was downloaded, and only the protein information included in the OXPHOS protein set was extracted and used for subsequent analysis. Searching and editing of gene symbols and selection of proteins were performed using Python software (v3.10.12).

For large-scale molecular docking, the collected SF compounds and proteins belonging to the OXPHOS protein set were preprocessed into a form that can perform docking analysis. Both the sdf compound and pdb protein files were converted into a pdbqt file format using the python openbabel library [56] (v3.1.0). To perform statistical processing of the docking results based on large-scale rigid docking analysis, both compounds and proteins were converted to rigid forms in the preprocessing step, and all torsion trees were removed.

Large-scale molecular docking analysis was performed using the python AutodockVina library [57] (v1.2.0). Rigid docking analysis was performed on all possible pairs of SF compounds and OXPHOS protein sets. The docking parameters were set to center = (0, 0, 0), box size = 126, and exhaustiveness = 100, and n_poses was set to 5, and the most optimal position among the five docking analyses was selected as the interaction position. The docking results were divided into strong hydrophilicity/weak hydrophilicity/weak hydrophobicity/strong hydrophobicity, and statistical processing was performed on each group. The mean, variance, and skewness for each group were calculated, and the distribution of the docking analysis data was visualized as a histogram (bins = 30) and Kernel Density Estimation (KDE) plot for each group. The statistical analysis of the docking analysis results was performed using the python scipy.stats library (v1.11.2), and the visualization was performed using the python seaborn library (v0.13.2).

### 2.8. OXPHOS PPI Network Construction and Analysis

The OXPHOS PPI network was constructed using PPI information provided by the STRING database [58] (v12, Obtained on 5 March 2024). Proteins included in the OXPHOS protein set were used as nodes, and the interaction scores between nodes were used as edges. The edge threshold was set to exceed 900, so that only connections with strong interactions were used. The constructed network was clustered into groups based on distance using the Louvain algorithm [59]. The network was constructed and clustered using the python NetworkX library [60] (v3.2.1), and the seed for Louvain community analysis was fixed to 0 to ensure the reproducibility of the study. The network was visualized using cytoscape software [61] (v3.10.1).

The proteins belonging to the groups divided by the Louvain algorithm were manually curated in the UniProt database to confirm which mitochondrial complex they belong to, thereby selecting the complex represented by each group [62]. Then, over-representation analysis (ORA) was used to confirm whether the groups clustered in the PPI network could represent the complex, and the unique physiological function of each group was identified. ORA was performed on the EnrichR platform (https://maayanlab.cloud/Enrichr/, accessed on 5 March 2025), using the results of the “Gene ontology (GO) Biological Process 2025 library” [63]. The five terms with the lowest *p*-values for each group were selected as the valid biological processes for each group, and the bar graph using this was downloaded from the analysis result graph provided by the EnrichR platform.

Using the docking analysis results of OXPHOS proteins and SF compounds, we predicted the pharmacological targets that are more likely to be significantly affected by groups of compounds with strong hydrophilicity, representative of the WESF, and strong hydrophobicity, which are difficult to find within the WESF. First, all docking results were collected by four hydrophilic groups; among them, the top 10% (*p* < 0.1) with the highest interaction potential for each group were selected as the main interaction results. Then, the proteins included in the main interaction results were counted to select the main action proteins for each group. As the number of compounds differs by group, the number of counted proteins was divided by the number of compounds to convert it into a function ratio. The major protein interaction ratio information calculated by the above method was used to distinguish between strong hydrophilic and strong hydrophobic function points in the OXPHOS PPI network. Proteins whose protein interaction ratio in the strong hydrophilic group was >0.05 compared with that in the strong hydrophobic group were selected as proteins with a high probability of interacting with the strong hydrophilic group. Conversely, proteins whose protein interaction ratio in the strong hydrophobic group was greater than 0.05 compared with that in the strong hydrophilic group were selected as proteins with a high probability of interacting with the strong hydrophobic group. Each selected protein was mapped to a network with color to identify the interaction point of each group, and groups with a high probability of acting on strong hydrophilicity were selected. The calculation of the major protein interaction ratio was performed using Python software (v3.10.12), and the visualization of major proteins was performed using Cytoscape software (v3.10.1).

### 2.9. Validation Using qPCR

qPCR analysis was performed to validate the results of transcriptome and docking analysis. We selected genes whose expression levels were reduced in the results of OXPHOS-related transcript expression using WESF and proteins with a high probability of interacting with the strong hydrophilic group in docking analysis. SW1783 cells (1.5 × 10^5^) were plated in a 6-well culture plate (Thermo Fisher Scientific) and incubated for 24 h. Then, the cells were treated with SF extracts and incubated for an additional 24 h. After washing the cells with ice-cold PBS, total RNA was prepared using an Easy-Spin^TM^ Total RNA Extraction Kit (#17221, iNtRON Biotechnology, Seongnam, Republic of Korea) following the manufacture’s guide. The concentration of total RNA was determined using a NanoDrop 2000 spectrophotometer (Thermo Fisher Scientific). First-strand cDNA was synthesized using 500 ng of total RNA and random primers using a High-Capacity cDNA Reverse Transcription Kit (#4368814, Thermo Fisher Scientific) following the manufacture’s guide. qPCR was performed using 25-fold diluted cDNA in ultrapure water, 250 μM gene-specific primer pairs (Genotech, Daejeon, Republic of Korea), and 1× Power SYBR^TM^ Green PCR Master Mix (#4367659, Thermo Fisher Scientific) in the CFX^TM^ Real-Time PCR System (Bio-Rad, Hercules, CA, USA). The sequences of primers used for qPCR were summarized in Table 1. Gene expression was calculated by the 2^−ΔΔCt^ method and normalized with respect to the housekeeping *GAPDH*. Relative gene expression in cells treated with SF extracts was determined with respect to that in vehicle (0.1% DMSO)-treated control cells.

### 2.10. Docking-Based DTI Network Construction and Analysis

Using the DTI results derived from the docking results, we constructed docking-based DTI networks to confirm the difference in the action point according to the degree of hydrophilicity. First, we constructed a network for each group using the major interaction results (*p* < 0.1) for each group selected above. The compounds and major action proteins for each group became nodes, and the interactions between the compounds and proteins were constructed as edges. After constructing the DTI network, we counted proteins belonging to the group (OXPHOS PPI group 2) that are likely to act on strong hydrophilicity in the OXPHOS PPI network analysis results to how well the DTI network according to hydrophilicity correlated with the PPI network analysis results. The group network was visualized using Cytoscape software (v3.10.1).

Centrality analysis was performed to determine the importance of the module proteins (OXPHOS PPI group 2) counted in the above method. First, we calculated the degree of each protein predicted to both belong to OXPHOS PPI group 2 and act on the DTI network, but to identify proteins that act more specifically for each DTI network. The degrees of the counted proteins were visualized as a heatmap. To emphasize the effect of strong hydrophilicity, we visualized only compounds wherein the degree of strong hydrophilicity is greater than the degree of strong hydrophobicity. Additionally, to determine how much each module protein group acts as a hub, we measured the proportion of edges that are module proteins among all edges in the network. Finally, to determine whether module proteins are connected to compounds that act as hubs, we computed NC and visualized it using a KDE plot. Most of the centrality calculations for the data were performed using Python software (v3.10.12). Neighborhood connectivity was performed using Cytoscape software (v3.10.1). Degree heatmap, edge ratio bar graph, and NC KDE plot were all constructed using the python seaborn library (v0.13.2).

### 2.11. Network Construction and Analysis Method Using PP

PP analysis, another bioinformatics method that can identify the differences between water and ethanol extracts, was used to predict the activation of OXPHOS by hydrophilic groups. PP analysis was performed using the well-known platform BATMAN-TCM [49]. Unlike version 2.0, version 1.0 allows downloading all interaction prediction scores; hence, we used BATMAN-TCM version 1.0 (http://bionet.ncpsb.org.cn/batman-tcm/index.php/Home/Index/index, accessed on 5 February 2025). Analysis was performed using the InChI of all collected SF compounds as queries, and the analysis results were downloaded and preprocessed by dividing them into PubChem CID, Gene Symbol, and interaction score. The preprocessed PP analysis results were classified into four groups according to hydrophilicity, and the thresholds of the interaction scores were set differently for each group to ensure consistency in data analysis. By selecting proteins with interaction scores of 10, 20, and 25 or higher for each group as valid DTIs, the number of valid proteins was set differently for each dataset. Among the 12 datasets classified into 4 groups and 3 thresholds, only proteins with 5 or more interactions were selected as valid to prevent cases predicted to interact by chance. Valid proteins were subjected to ORA on the EnrichR platform using Gene symbol information (accessed on 5 March 2025). After downloading all ORA results analyzed using the KEGG 2021 Human set, the adjusted *p*-value of the “Oxidative Phosphorylation” term in each valid protein dataset was used. For data sets where OXPHOS was analyzed as a non-significant pathway in the ORA results, the adjusted *p*-value was set to 1. To confirm the tendency for OXPHOS activity regulation in a valid protein dataset, the −log_10_(adjusted *p*-value) value was used to visualize the bar graph, and the proteins predicted to affect OXPHOS activity for each valid protein dataset were merged and displayed as a heatmap. The adjusted *p*-value bar graph and OXPHOS protein heatmap were visualized using the python seaborn library (v0.13.2).

In addition, to confirm how the importance of 17 proteins predicted to affect OXPHOS activity in PP analysis changes according to the difference in hydrophilicity, PP DTI network (PDN) was constructed, and the action points of the 17 proteins were identified. PDN used four hydrophilicity group datasets with a threshold set to 10. Proteins interacting with SF compounds belonging to each hydrophilicity group were set as nodes, and connections with an interaction score of 10 or higher were set as edges. The network was constructed using compound spring embedder layout [64]—an algorithm in which nodes with large centrality are gathered in the center and spread outward if they have small centrality. The 17 OXPHOS-active proteins were mapped with different colors to enable observation of the centrality trend in the network, and NC was calculated in each network to confirm which hydrophilicity group is connected to important ligands. PDN construction, visualization, and NC calculation were performed using Cytoscape software (v3.10.1).

### 2.12. Docking–PP Integrated Network Analysis

To predict the importance of COX5B—a protein commonly derived as playing an important role in transcriptome analysis, docking analysis, and PP analysis—a DTI integrated network was constructed. First, the docking-based DTI network and PDN were integrated for each hydrophilicity group. Then, the network was preprocessed in two ways to confirm the action pattern of COX5B. First, we built an FRN to confirm the hub characteristic of COX5B in the OXPHOS protein set. In this network, we used a protein set containing OXPHOS PPI network group proteins (OXPHOS PPI group 2) and 17 OXPHOS-active proteins used in PDN analysis. The network was reconstructed by setting only the SF compounds directly connected to the OXPHOS PPI group 2 proteins in the DTI network and the SF compounds directly connected to the 17 OXPHOS active proteins in the PP network as nodes. The second method was used to determine the extent to which the SF compounds act as a major target of COX5B. The SRN is the firstshell network of the FRN, and the network was reconstructed using all proteins predicted to interact with SF compounds in the FRN nodes. The protein nodes connecting the compounds were extracted from the DTI network and the PP network, respectively. The network reconstruction process was performed using Cytoscape software (v3.10.1).

To determine the centrality of COX5B in the FRN and SRN based on the four hydrophilicity groups, we performed centrality analysis. For network-based centrality analysis, degree centrality, betweenness centrality, closeness centrality, PageRank centrality, and harmony centrality methods were used, respectively [65]. To identify the tendency of centrality with different ranges after the analysis, a max–min normalization process was performed to set the largest value to 1 and the smallest value to 0 for each centrality analysis result [66]. To determine whether COX5B has different centrality values for each degree of hydrophilicity, the centrality values of COX5B in each network were visualized as a bar graph. In addition, to confirm how important a role COX5B plays in the overall network, the centrality value of the overall network is presented as a box plot, and the centrality value of COX5B is shown to confirm the hub property of COX5B. Both the bar graph and the box plot were visualized using the python seaborn library (v0.13.2).

### 2.13. In Vitro OXPHOS Activity Assessment

ATP, a crucial energy source for living cells, is synthesized by glycolysis and mitochondrial OXPHOS. To investigate the differential effects of SF extraction methods on energy metabolism, intracellular ATP was quantified in SW1783 treated with WESF or EESF in the presence and absence of oligomycin treatment. The cells (5 × 10^3^) were plated in 96-well plates and incubated 24 h. The culture medium was replaced with fresh growth medium containing SF extracts and incubated for an additional 24 h. Then, the culture medium was replaced again with a fresh growth medium containing a combination of SF extracts and 2 mM oligomycin (O4876, Sigma-Aldrich, St. Louis, MO, USA). After 6 h, the cells were washed with ice-cold PBS and lysed for 5 min with shaking at 700 rpm in 50 mL of lysis buffer (100 mM Tris, pH 7.5, 4 mM EDTA, 0.5% TCA, 0.1% TX-100. The intracellular ATP contents and the lactate concentration in the culture medium were determined using a PromoKine ATP Quantitation Kit (PK-CA577-K354, PromoCell GmbH, Heidelberg, Germany) following the manufacturer’s instructions.

### 2.14. In Vitro Antioxidant Activity Assessment

The antioxidant activities of SF extracts were compared in the H_2_O_2_-stimulated SW1783 cells according to their extraction solvents. Briefly, cells (5 × 10^3^) were plated in 96-well plates and incubated 24 h. The growth medium was then replaced with fresh medium containing WESF (20, 100, 500 μg/mL) or EESF (12.4, 62, 310 μg/mL) for 24 h. After washing the cells with growth medium, they were exposed to medium containing 0.25 mM H_2_O_2_. After a 6 h incubation, the cells were washed with HBSS and incubated in growth medium containing CellROX Green (5 μM) for an additional 30 min. Following three washes with HBSS, the fluorescent intensity was measured at excitation 485 nm/emission 520 nm using a SpectraMax i3 microplate reader (Molecular Devices, Sunnyvale, CA, USA).

### 2.15. Statistics

Statistical analyses were performed using GraphPad Prism (v.7.05, GraphPad, San Diego, CA, USA). In vitro data such as qPCR and ATP energy metabolism assays are presented as mean ± standard deviation (SD) of three replicates. The means were compared using two-way analysis of variance followed by post hoc multiple comparisons test. The mean difference was considered significant at *p* < 0.05.

## 3. Results

### 3.1. Transcriptome Analysis of WESF and EESF

**Figure 1:**
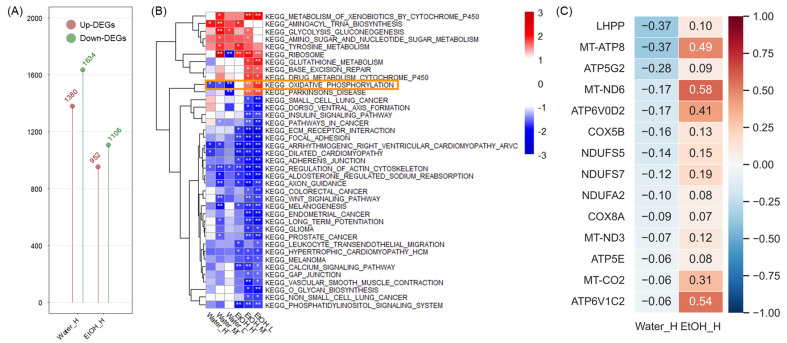
Transcriptome analysis results of WESF and EESF in SW1783 cell line. (**A**) The results of DEGs analysis derived from WESF and EESF, respectively. Up-DEGs indicate overexpressed transcriptomes, and Down-DEGs indicate under-expressed transcriptomes. (**B**) GSEA results using transcriptome expression results of WESF and EESF. The orange box highlights the OXPHOS pathway, which has the greatest tendency for activity to be reversed in WESF and EESF. (**C**) Comparison of the expression of transcripts involved in the OXPHOS pathway. Heatmap showing the log_2_FC values of genes matching the direction of OXPHOS pathway activation in WESF and EESF. WESF, hot water extract of Schisandrae Fructus; EESF, ethanol extract of Schisandrae Fructus; DEGs, differentially expressed genes; GSEA, Gene set enrichment analysis; OXPHOS, oxidative phosphorylation; FC, fold change. * *p* < 0.05, ** *p* < 0.01.

DEG analysis of the two extracts revealed a greater number of DEGs in the WESF than in the EESF (Figure 1A). In the high-dose WESF group, there were 1380 upregulated DEGs and 1634 downregulated genes (Supplementary Table S1). Meanwhile, in the high-dose EESF group, there were 952 upregulated and 1106 downregulated genes (Supplementary Table S2).

GSEA showed similar KEGG pathway enrichment between WESF and EESF. However, ribosome, OXPHOS, and Parkinson’s disease terms exhibited opposite enrichment between the two extracts at least at one concentration (Figure 1B). In particular, the OXPHOS pathway showed a completely opposite trend in WESF and EESF.

Genes with opposite expression in the OXPHOS pathway were identified from 133 proteins selected as part of the OXPHOS pathway gene set (Supplementary Table S3). Among them, 15 genes exhibited contrasting expression between the high-dose WESF and EESF (Supplementary Tables S1 and S2)—among which, *UQCR11* was excluded from the analysis because its amino acid sequence matched only 84% in the GeneCards database search, resulting in 14 genes being selected (Figure 1C).

### 3.2. SF Compounds

The total number of SF compounds collected from 3 natural product databases and whose 3D structures were searched in the PubChem database was 441 (Supplementary Table S4). The XLogP values of the compounds used as key features in this study ranged from −8.0 to 10.9, and included both hydrophobic and hydrophilic properties.

When classifying the hydrophilicity of SF compounds into quartiles, many compounds were included in the borderline between classifications; therefore, the XLogP value that could best match the number between groups was selected. The XLogP values used to assess degree of hydrophilicity were as follows: Strong hydrophobic group (Spho) > 4.9, Weak hydrophobic group (Wpho) > 4, Weak hydrophilic group (Wphi) > 2.3, and those below that were classified as Strong hydrophilic group (Sphi). This classification revealed 104 strong hydrophobic compounds, 108 weak hydrophobic compounds, 118 weak hydrophilic compounds, and 111 strong hydrophilic compounds in each group, respectively (Supplementary Table S4).

### 3.3. Large-Scale Molecular Docking Based on OXPHOS-Related Proteins

All 441 compounds of SF and 133 OXPHOS proteins were preprocessed in pdbqt form for docking analysis (Supplementary Materials S1 and S2). The results of docking analysis using the preprocessed compound–protein pairs were classified into each hydrophilicity group, and descriptive statistics were analyzed (Supplementary Materials S3). Sphi showed the lowest average docking scores, indicating that it was the group with the highest possibility of interaction (Supplementary Figure S1). Meanwhile, the variance was also the highest in Sphi, indicating the greatest diversity of drug interactions. The absolute value of skew was also the highest in Sphi, suggesting that it included targets with extremely high potential for interaction.

### 3.4. OXPHOS PPI Network

**Figure 2:**
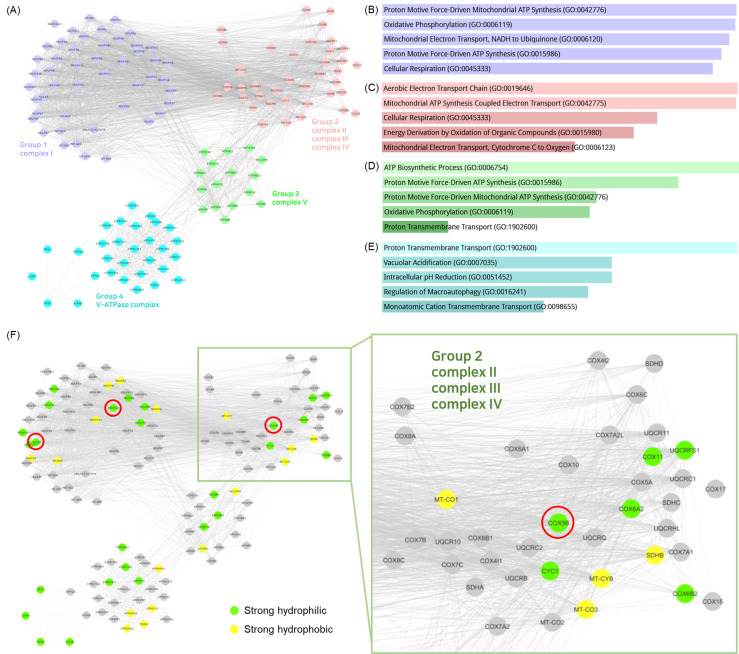
Results of OXPHOS PPI network construction and analysis. (**A**) PPI network of proteins involved in the OXPHOS pathway. Each group was classified using the Louvain algorithm, and color-mapped with different colors for group classification. (**B**–**E**) ORA results analyzing the biological process most closely related to OXPHOS PPI groups 1–4, respectively. The longer the bar graph and the darker the color, the smaller the *p*-value. (**F**) The action points of the SF compounds using docking analysis. Green nodes are proteins with which strong hydrophilic compounds are likely to interact, and yellow nodes are proteins with which strong hydrophobic compounds are likely to interact. Red circles indicate genes that match the expression direction of OXPHOS pathway activity of WESF in Figure 1C. OXPHOS, oxidative phosphorylation; PPI, protein–protein interaction; ORA, over-representation analysis; WESF, hot water extract of Schisandrae Fructus.

The OXPHOS PPI network was constructed with 132 protein nodes and 2928 edges (Figure 2A, Supplementary Table S5). Clustering the network modules using the Louvain algorithm divided the network into four groups (Supplementary Table S6). The modules represented respiratory complex I (group 1), respiratory complex II & III & IV (group 2), respiratory complex V (group 3), and V-ATPase complex (group 4) (Supplementary Table S7). The ORA results indicated that group 1 plays a role in proton motive force-driven mitochondrial ATP synthesis (Figure 2B); group 2 is involved in aerobic electron transport chain (Figure 2C); group 3 was most closely related to the ATP biosynthetic process (Figure 2D); and group 4 was implicated in proton transmembrane transport, aligning with the functions of respective complexes (Figure 2E).

### 3.5. qPCR Validation for Candidate Prioritization

We performed qPCR experiments to select a protein candidate that should be prioritized among the multiple candidates identified. The selected protein was subsequently used as the key candidate target for interpretation in the following in silico analyses. Analysis of major protein interactions derived from the OXPHOS PPI network and docking analysis indicated that 26 proteins were highly likely to interact with Sphi, while 14 proteins were highly likely to interact with Spho (Figure 2F). In groups 1–3, proteins with a high possibility of interacting with Sphi were 1.5 times more likely to interact than were those with Spho. NDUFS5 and NDUFS7 in group 1 as well as COX5B in group 2 showed the same trend as that noted in transcript expression results (Figure 1C and Figure 2F). qPCR analysis revealed that NDUFS5 and NDUFS7 consistently showed downregulated expression levels with increasing concentrations, regardless of solvent type (Supplementary Figure S2). In contrast, COX5B displayed differential expression patterns between the two solvent conditions as concentration increased, with statistically significant differences observed at higher concentrations between WESF and EESF. Thus, it was predicted that group 2 contained proteins that could best explain OXPHOS-related gene expression changes.

### 3.6. Docking-Based DTI Network

**Figure 3:**
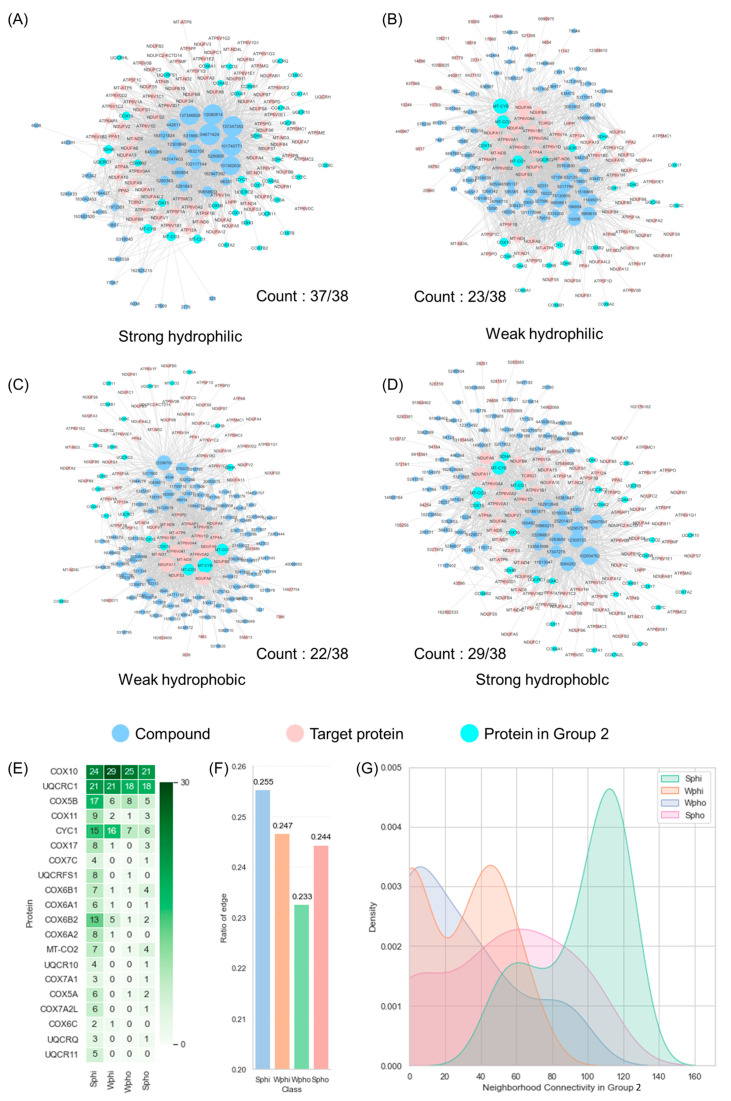
Results for constructing and analyzing docking-based DTI network. (**A**–**D**) Docking networks of SF compounds and proteins constituting the OXPHOS pathway. Blue nodes are SF compounds, pink nodes are OXPHOS proteins, and only the nodes that were mainly analyzed in the docking results are visualized. (**A**–**D**) Networks using Sphi, Wphi, Wpho, and Spho compounds, respectively. Cyan nodes are the action points of group 2 selected in Figure 3F, and the count value indicates how many of the 38 group 2 proteins were included in each DTI network. (**E**) The number of degrees of proteins with a higher degree in Sphi than in Spho among the major proteins in group 2 is visualized as a heatmap. (**F**) The number of edges that group 2 nodes have compared to the total number of edges in each network is represented as a bar graph. (**G**) Kernel density estimation of the value of neighborhood connectivity that group 2 nodes have. DTI, drug–target interaction; SF, Schisandrae Fructus; Sphi, strong hydrophilic group; Wphi, weak hydrophilic group; Wpho, Weak hydrophobic group; Spho, strong hydrophobic group.

DTI networks were constructed according to the hydrophilicity (Table 2 and Figure 3A–D, Supplementary Table S8). The number of Sphi compounds predicted to interact was the lowest at 39, but the number of interacting proteins was relatively high, at 131. In contrast, the other three groups had many compound nodes; although Spho protein nodes were the highest at 132, the number of interacting proteins per compound was confirmed to be less than that of Sphi. In group 2, 37 proteins in Sphi, 23 proteins in Wphi, 22 proteins in Wpho, and 29 proteins in Spho, which were predicted to best explain OXPHOS-related expression changes, were included in the DTI network; their interaction with SF compounds was further analyzed.

### 3.7. Centrality Analysis of Valid Protein Groups Derived from Docking

The degree analysis of proteins belonging to OXPHOS PPI network group 2 identified proteins with high interaction potential in each group (Supplementary Table S9). For example, MT-CO1 frequently interacted with Wpho, COX15 with Wphi, and SHDB with Spho. Among them, Sphi frequently interacted with COX11, COX17, COX6A1, COX6B2, COX6A2, COX7A2L, and UQCR11, and was predicted to be the group with the highest interaction potential. Among them, COX5B interacted with 17 of the 39 candidate compound nodes, confirming that it was one of the important proteins in the within- and between-group comparisons (Figure 3E). In addition, to determine the centrality index of the major group proteins in the network, we calculated the proportion of edges for proteins belonging to group 2 of the OXPHOS PPI network in each DTI network (Supplementary Materials S4) Supplementary Data S4. Sphi had the highest value among the four hydrophilicity groups, with 25.5% of the total edges connected to the major proteins (Figure 3F). Finally, we performed neighbor connectivity analysis to determine whether proteins belonging to group 2 were connected to important nodes in the network (Supplementary Table S10). The KDE plot showed that other hydrophilicity groups were more likely to have no neighborhood connectivity (NC) or data distributed around 60, while only Sphi was more likely to have many data in the NC around 110 (Figure 3G).

### 3.8. Network Construction Using PP

**Figure 4:**
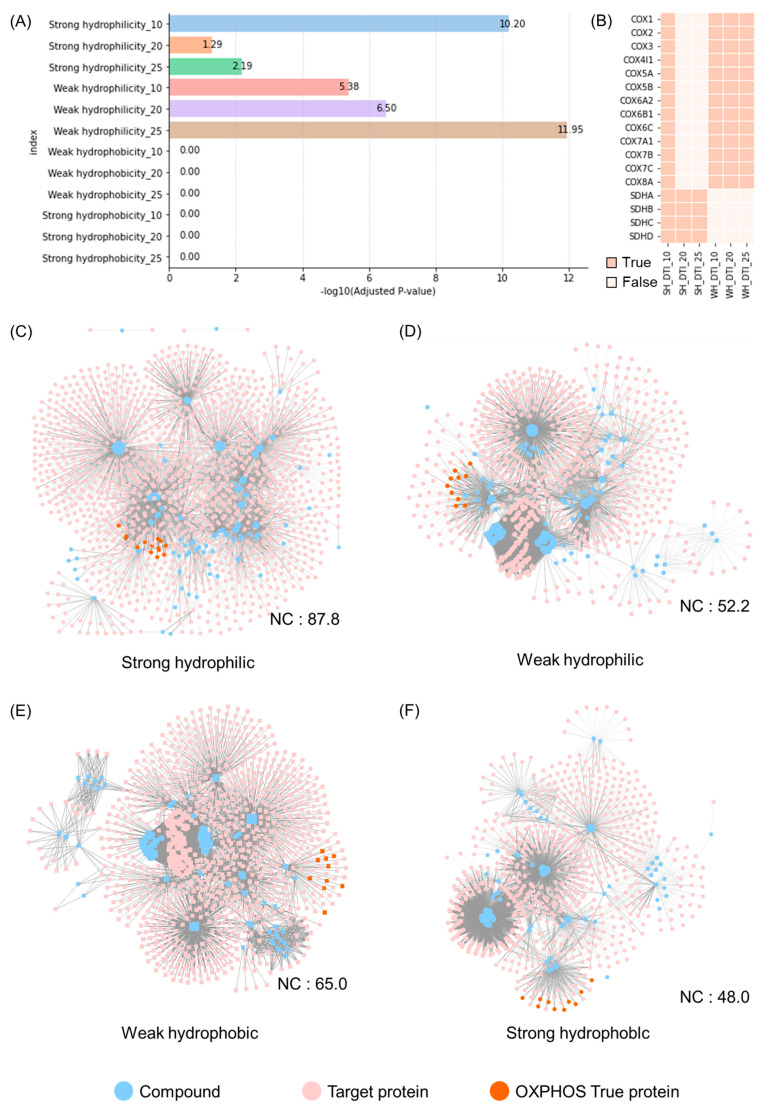
Network construction and analysis results using PP. (**A**) The results of ORA performed using the PP analysis results are represented as a bar graph. The length and score of the bar graph represent the log value of the adjusted *p*-value of each group. (**B**) Proteins in the groups that showed significant *p*-values in the PP analysis results are visualized as a heatmap. (**C**–**F**) Interactions mainly derived from the PP results are classified by hydrophilicity and visualized as a DTI network. Blue nodes are SF compounds, and pink nodes are interacting proteins. Red nodes labeled as OXPHOS True proteins are 17 proteins derived from (**B**). NC is the average of the neighbor connectivity values of OXPHOS True proteins. PP, polypharmacology; ORA, over-representation analysis; DTI, drug–target interaction; OXPHOS, oxidative phosphorylation.

PP analysis enabled assessment of 8,394,876 interactions for 441 SF compounds and 19,036 proteins. The analysis results were classified by hydrophilicity group, and interactions with scores of 10, 20, and 25 or higher were selected as valid and preprocessed (Supplementary Materials S5). We analyzed 286, 188, and 64 proteins with interaction scores of 10, 20, and 25 or higher and 5 or more hits in Sphi. Under the same conditions, 352, 263, and 92 proteins were predicted in Wphi vs. 209, 167, and 60 proteins in Wpho, and 370, 347, and 132 proteins in Spho. OXPHOS ORA results using the KEGG gene set in the above-predicted 12 groups showed that all hydrophilic groups significantly affected OXPHOS, whereas none of the hydrophobic groups were significant (Figure 4A). A total of 17 proteins affected OXPHOS, and Sphi was predicted to affect SDH protein, while Wphi was predicted to affect COX protein levels (Figure 4B). Among them, Sphi with an interaction score of 10 or higher included both SDH and COX proteins.

A PP DTI network was constructed for each hydrophilicity group (Supplementary Materials S6), and its characteristics are shown in Table 3. The number of Sphi compounds predicted to interact was 88, but the number of interacting proteins was relatively high at 1459. In contrast, in the three other groups, the number of proteins analyzed was less than that of Sphi, compared with the compounds, showing a tendency similar to the docking-based DTI network (Figure 3A–D). The above-constructed network was visualized by changing the network layout using the compound spring embedder (Figure 4C–F). In particular, when the 17 proteins that act on OXPHOS were marked in red to confirm their location in the network topology, they were relatively densely concentrated in the center in Sphi, while they spread outward in the other three groups, confirming the tendency for OXPHOS proteins to play an important role only in Sphi (Figure 4C). In addition, to quantitatively evaluate centrality, we calculated the NC that could be analyzed in the bipartite network, revealing the average NC of the OXPHOS proteins in the Sphi group to be 87.8—which was relatively high compared with that of other networks. Thus, OXPHOS proteins have a more central location or function in the Sphi network than in other networks.

### 3.9. Docking–PP Integrated Network

**Figure 5:**
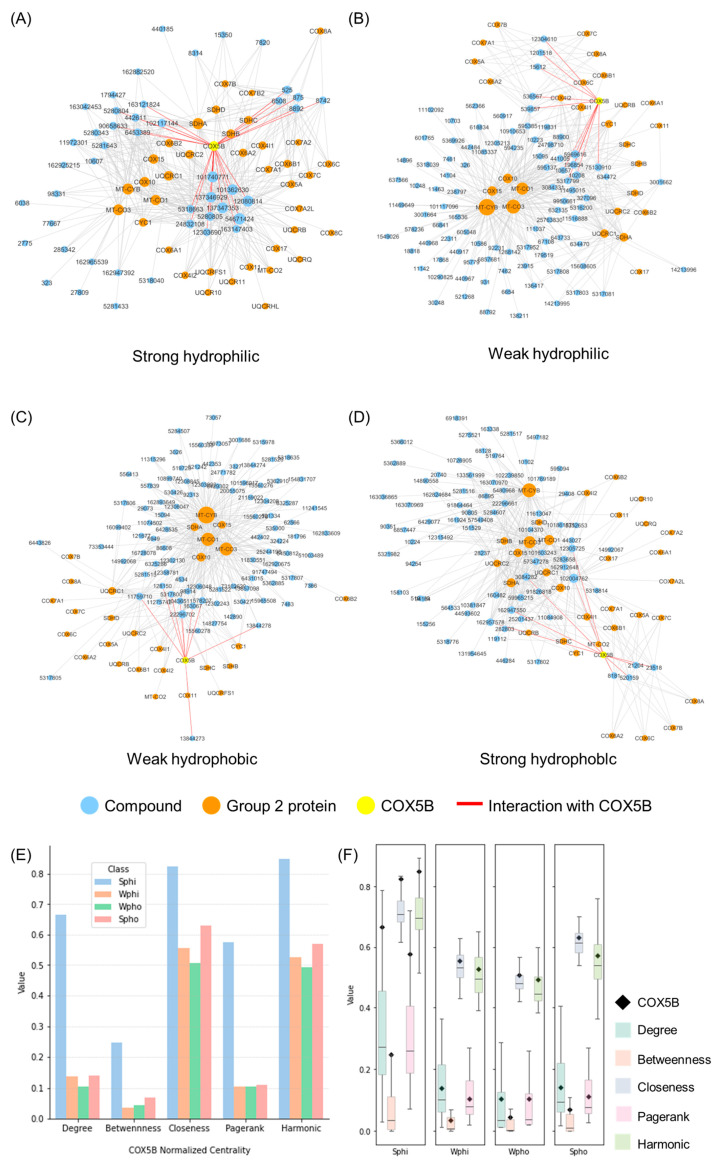
Docking–PP integrated network analysis results. (**A**–**D**) Network reconstructed using the major proteins of the docking-based DTI network and the PP DTI network. The two networks were integrated for each group classified based on hydrophilicity using the compounds connected to the major proteins. The blue nodes are SF compounds, and the orange nodes are group 2 proteins, which are the major proteins selected in Figure 3F. The yellow node is COX5B, and the red line is a component predicted to interact with COX5B. (**E**) The result of calculating the centrality value of COX5B in the (**A**–**D**) network. (**F**) The result of calculating the relative centrality distribution of COX5B in the (**A**–**D**) network. The box plot shows the distribution of the centrality values of all nodes in the network, and the diamond-shaped dots indicate the location of COX5B. PP, polypharmacology; DTI, drug–target interaction.

To integrate transcriptome, docking, and PP analysis, we constructed a first reconstructed network (FRN) (Figure 5A–D, Table 4, Supplementary Materials S7), and confirmed the central index of COX5B. Sphi had the fewest nodes, at 83, but the most edges, at 451 (Figure 5A). In each network rearranged by the compound spring embedder algorithm, COX5B was located at the center only in Sphi, while being in the periphery of the other three networks. The degree of COX5B was 22 in Sphi, 11 in Wphi, 9 in Wpho, and 9 in Spho. Sphi had the largest number of edges. Five centrality values calculated using other centrality algorithms, including degree, also showed that COX5B had a higher centrality in Sphi than in the other groups (Figure 5E). In addition, to determine the relative centrality of COX5B in the network, we compared it with the centrality values of other proteins that composed the network. Consequently, Sphi showed higher centrality, compared with the box plot, suggesting that Sphi plays a relatively important role in the network (Figure 5F, Supplementary Materials S8). In other groups, COX5B showed higher centrality than the average, but this difference was not considerable. This suggests that in other groups, COX5B plays a relatively less important role compared with that in Sphi.

Following FRN construction, we constructed second reconstructed network (SRN) to confirm whether compounds specifically act on COX5B according to polarity (Supplementary Figure S3A–D, Supplementary Materials S9). The number of nodes in Sphi was 841, which was slightly more than that in the Spho group, at 830. However, the number of edges was 2776, approximately twice as much as that in the Spho group, at 5322 (Supplementary Table S1, Supplementary Materials S10). In the centrality analysis of COX5B, all centrality scores were higher in Sphi than in other groups; in particular, the closeness network showed a high score exceeding 0.8 (Supplementary Figure S3E). In addition, the relative centrality of COX5B in the network confirmed that it was located at a very high rank in Sphi, confirming that COX5B is a core protein in Sphi (Supplementary Figure S3F).

### 3.10. Effect of WESF and EESF on OXPHOS and Antioxidant Activities

Glycolysis/OXPHOS assays probe the glycolytic capacity of cells under specific culture conditions. Oligomycin inhibits mitochondrial ATP synthesis by binding to the proton channel on the F_0_ component of ATP synthase [67]. Consequently, in the presence of oligomycin treatment, the cells rely entirely on glycolysis to produce ATP energy. Therefore, total ATP is the sum of ATP produced through glycolysis and ATP generated through OXPHOS. The energy metabolic shift was investigated in SW1783 cells treated with WESF and EESF, both in the presence and absence of oligomycin co-treatment.

**Figure 6:**
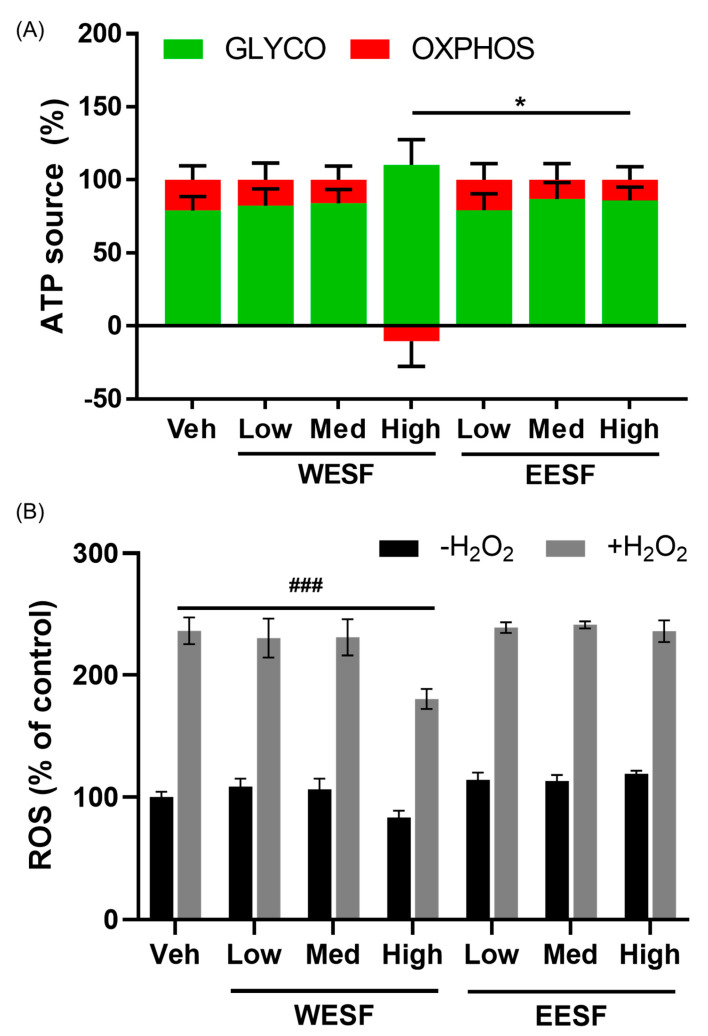
Differential effects of SF extracts on energy metabolism and oxidative stress. (**A**) The relative energy source of ATP was determined in SW1783 cells treated with a combination of SF extracts and oligomycin co-treatment. (**B**) Intracellular ROS level was measured in SW1783 cells in the absence and the presence of H_2_O_2_ stimulation. SF, Schisandrae Fructus; WESF, hot water extract of SF; EESF, ethanol extract of SF. Low (20 µg/mL), medium (100 µg/mL), high (500 µg/mL) for WESF. Low (12.4 µg/mL), medium (62 µg/mL), high (310 µg/mL) for EESF. * *p* < 0.05, ### *p* < 0.001.

In SW1783 cells treated with WESF, OXPHOS decreased in a dose-dependent manner. Notably, at a high dose of WESF, OXPHOS was completely inhibited, and ATP was produced exclusively through the glycolytic pathway. In the case of EESF treatment, SW1783 showed a slight decrease in OXPHOS. However, ATP production via OXPHOS persisted even at high doses of EESF (14% of total ATP), highlighting the differential effects of SF extracts based on solvent extraction methods (Figure 6A).

Assuming that a shift in cellular energy metabolic may regulate intracellular oxidative stress, the antioxidant activity of SF extracts was evaluated according to the solvents. In the absence of H_2_O_2_ stimulation, WESF, but not EESF, marginally reduced basal ROS level. Exposure to H_2_O_2_ increased the intracellular ROS levels by approximately 230%, which was significantly attenuated by WESF at higher concentration. In contrast, EESF failed to suppress H_2_O_2_-induced ROS generation. Under basal conditions without H_2_O_2_ stimulation, neither WESF nor EESF altered intracellular ROS level (Figure 6B).

## 4. Discussion

In this study, we aimed to evaluate the differential influence on OXPHOS activity of compounds contained in SF, based on polarity. To this end, we predicted mechanistic differences between water and ethanol extract using multiple bioinformatics analysis methods, and verified the differences in activity through in vitro experiments. The maximum concentration for WESF (500 µg/mL) and EESF (310 µg/mL) were selected as the maximum tolerable concentrations to evaluate the efficacy and mechanisms of the extracts in vitro without any confounding toxic effects. Unlike single compounds, WESF and EESF are multi-component crude extracts consisting largely of major primary metabolites alongside minor active phytochemicals. Therefore, the concentrations of biologically active constituents are expected to be substantially lower than the total extract concentrations, and this should be considered when interpreting the physiological relevance of the in vitro concentrations. By quantifying the transcriptome of cells treated with WESF and EESF, we demonstrate differences in the effects on OXPHOS activity (Figure 1). To determine the reason for these differences, OXPHOS PPI network and docking analysis were performed to predict the characteristics and targets of extract compounds with strong hydrophilicity (Figure 2, Supplementary Figure S1). Thereafter, docking and PP-based DTI network analysis were performed to predict respiratory complex II & III & IV as targets of Sphi compounds. More specifically, COX5B was discovered as a key target (Figure 3 and Figure 4, Supplementary Figure S2). Through docking–PP integrated network analysis, we confirmed a central role for COX5B (Figure 5, Supplementary Figure S3). Finally, through in vitro experiments, we confirmed that COX5B activity was reduced and that ATP production via OXPHOS was inhibited by the WESF (Figure 6, Supplementary Figure S2).

Our study was conducted in two major stages. In the first stage, we performed a progressive narrowing process to identify candidate proteins that were likely to be involved in the activity of WESF (Figure 1 and Figure 2). Through transcriptomic analysis, we identified a pathway that was differentially regulated between the water extract and ethanol extract. Based on this pathway, we subsequently used public database-based network analysis, exploratory docking analysis, and qPCR validation to identify COX5B as a key candidate protein. Rather than starting from a predefined target protein, this top-down approach of gradually narrowing candidate targets has the advantage of reflecting system-level biological changes while identifying key regulatory factors in natural product-based research. Because biological changes induced by complex herbal extracts are difficult to explain using only a single target-based approach, this top-down strategy may represent a useful approach for mechanistic exploration. In the second stage, we aimed to verify that the identified key candidate protein was not selected coincidentally by validating the selective OXPHOS inhibition and ROS suppression observed in WESF through multiple independent network analyses and in vitro experiments (Figure 3, Figure 4, Figure 5 and Figure 6). Computational biology analyses have the advantage of generating new hypotheses and research insights based on previously accumulated biological data. However, they also have the limitation that the interpretation of the results can vary depending on the parameters and analytical strategies used. Therefore, verifying consistency across independent analyses or using multiple analytical parameters is important for improving analytical reproducibility and robustness [68,69]. Through docking analysis, network analysis, and an integrated analysis combining both approaches, we consistently identified COX5B as a key regulator of OXPHOS, which strengthened the reliability of our interpretation. In addition, the final in vitro experiments also demonstrated consistent patterns. This approach provides an example of how integrating independent computational analyses can improve the consistency and interpretative reliability of the results, and may serve as a useful strategy for future computational biology-based mechanistic studies.

DEG analysis, using the SW1783 cell line, revealed that the number of DEGs in the WESF group was higher than that in the EESF group (Figure 1A). In a previous study that used the same operating procedure to obtain transcriptomes, the number of DEGs was higher in the ethanol extract for all 10 herbs, 4 decoctions, and 4 cell lines (A549, HepG2, HT29, SW1783) [70]. However, the opposite result was noted only in SF-treated SW1783 cells. This suggests that SF, unlike other herbs, may have the potential to treat the nervous system when used as WESF, as shown in previous studies [25]. In particular, the expression levels of the OXPHOS genes were different between the WESF from the EESF groups (Figure 1B,C). The tendency toward lower OXPHOS activity is consistent with the efficacy of SF, which has been traditionally used to suppress responses after excess exercising or high solar radiation exposure [1]. These multi-compound-based extract mechanism studies that utilized transcriptomic analyses represent an efficient research approach to identify the mechanisms of existing traditional treatments that have been used effectively.

In this study, we classified the compounds of SF based on hydrophilicity and performed large-scale molecular docking to predict the interactions of the compounds according to hydrophilicity (Supplementary Figure S1). The binding affinity of Spho was the lowest among the four hydrophilicity groups. This result is consistent with existing knowledge, as hydrophobic drugs are more likely to easily pass through the phospholipid bilayer, and their excretion process is slower than that of hydrophilic drugs [71]. However, the variance of binding affinity and the absolute value of skewness were the highest in Sphi. These results suggest that the diversity of drug interactions is the highest in Sphi, and there may be many compounds with very high interaction potential in Sphi. This pattern can also be confirmed through network analysis. Although the number of interacting compounds in Sphi was small, each compound interacted with a very large number of proteins (Table 2 and Table 3). In addition, OXPHOS PPI network group 2 analysis revealed the lowest number of compounds although the number of proteins was almost the same (Figure 2F, Table 4). These results suggest that even low XlogP compounds can act as potential drugs; further, in a PP-based approach, a specific hydrophilic compound may be the key compound with the best efficacy. We searched the previous literature to determine which compounds may be selectively extracted in water extracts by directly comparing WESF and EESF. However, to the best of our knowledge, such studies have not yet been performed. Ideally, the most robust approach would be to include both profiling and validation experiments. However, due to practical limitations in available resources, we considered how the compositional differences between WESF and EESF could be reflected as much as possible and therefore performed computational modeling by reusing compound information from public databases. Because it is practically difficult to perfectly reproduce the complete chemical composition of complex extracts, our study focused more on the relative physicochemical characteristics associated with water and ethanol extraction. Although this approach may be interpreted as a biological simplification in that it selectively emphasizes certain characteristics, such selective emphasis strategies are widely used in computational modeling studies [72]. For example, Ridge and LASSO methods used in machine learning selectively reflect important features by shrinking some coefficients or setting them to zero, which can reduce overfitting and improve the robustness of the model. In this study, we derived the core mechanistic insights based on the concept of selective emphasis, and our results suggest that this approach may represent a useful strategy in situations where data are limited. Nevertheless, the absence of chemical profiling remains an important limitation of this study. Therefore, future studies will be required to directly characterize the compositional differences between WESF and EESF using LC-MS/MS or metabolomic profiling-based analyses and to further validate the compound–target associations and mechanistic interpretations proposed in this study.

We used a multi-bioinformatics approach to predict the activity differences according to the hydrophilicity of SF compounds and discover targets. In the OXPHOS PPI network, we confirmed COX5B as a candidate target, supported through docking analysis results and qPCR validation (Figure 2, Supplementary Figure S2). In the docking-based DTI network, we confirmed that the Sphi had the most connections and NCs with OXPHOS PPI network group 2 (Figure 3). In particular, proteins such as COX5B, CYC1, and COX6B2 are most likely to interact with Spho compounds [73]. In the network analysis based on PP, only strong hydrophilic and weak hydrophilic groups were related to OXPHOS function. In particular, COX and SDH families were related (Figure 4). Although the conclusions derived from the PPI, docking DTI, and PP network were slightly different, they shared COX5B. Bioinformatics analysis methods have been steadily developed, yielding excellent prediction results [27]. However, they remain limited, in that, one method alone cannot explain the entire process of life phenomena. Therefore, utilizing diverse analytical methods to examine the same phenomenon from multiple perspectives offers a strategic advantage in understanding complex biological systems. In this context, multi-bioinformatics analysis plays an important role in in silico prediction experiments.

Based on the conclusions described above, we predicted that COX5B plays a key role in the OXPHOS inhibition by SF (Figure 5, Supplementary Figure S3). Among the multiple centrality indices, closeness was significantly higher than degree (Figure 5E). In the SRN, we confirmed that the closeness of Sphi was overwhelmingly higher than the degree (Supplementary Figure S3E). Closeness centrality is known as an indicator of distance from all nodes [74]. The relatively low degree centrality and high closeness centrality in Sphi suggest that all Sphi compounds do not necessarily act on COX5B, but are located at the center of the network, which may indirectly and efficiently connect with several other targets. This can be interpreted in the same context as the high NC of OXPHOS-related proteins in Figure 4. Although high NC does not guarantee a role as hubs, the very high centrality of the connected nodes suggests that they likely belong to the core network, which may facilitate indirect and efficient connections [73]. These results can be interpreted as COX5B acting as an efficient mediator in the OXPHOS pathway from a network topology perspective. Therefore, it can be explained that COX5B is more likely to interact with the Sphi than with other groups, which enables efficient functional inhibition.

This is consistent with the observations of previous studies highlighting the biological significance of COX5B—which was identified as a major target in the present study (Figure 1, Figure 2, Figure 3, Figure 4 and Figure 5)—as a cytochrome c oxidase complex accessory subunit [75]. It plays a role in ATP production, exhibiting high RNA expression in energy-consuming tissues such as the muscle [76]. COX5B has been studied extensively in relation to cancer. More specifically, its expression is known to increase in various cancer types, including breast cancer, glioma, hepatoma, and colon cancer [77,78,79,80,81]. High COX5B expression in tumors may affect the prognosis of cancer patients through enhanced angiogenesis, growth, and proliferation [77,81]. We predicted distinct COX5B expression patterns upon WESF and EESF treatment through multi-bioinformatics analyses, which was then verified through further experiments. COX5B expression was reduced in the WESF (Supplementary Figure S2), with OXPHOS-related expression also being reduced (Figure 6A). Additionally, we investigated whether the reduction in OXPHOS associated with COX5B suppression was linked to the regulation of ROS production. The experimental results showed that ROS levels were reduced only in the WESF-treated group, whereas no significant change was observed in the EESF-treated group (Figure 6B). These results indicate that WESF suppresses OXPHOS-associated ROS production. COX5B downregulation is known to reduce mitochondrial OXPHOS activity, resulting in decreased ROS production through reduced electron leakage. Based on these previously reported mechanisms, WESF suppresses ROS generation and contributes to anticancer effects as well as the prevention of excessive oxidative stress-induced cellular damage and aging [82,83]. Future studies should further validate the anticancer and aging inhibition mechanisms of WESF.

This study has some limitations. First, as mentioned above, due to limitations in our available equipment and personnel, we were unable to perform a comprehensive profiling analysis—to clarify previous reports suggesting that differences exist in composition between WESF and EESF. In addition, the classification of strong hydrophilic compounds was based on the characteristics of solvents that can only extract hydrophilic compounds. As ethanol extracts extract both hydrophilic and hydrophobic compounds, hydrophobicity alone could not be concluded. To overcome this issue, the study focused on hydrophilic compounds while excluding analysis and discussion of hydrophobic compounds as much as possible.

Nevertheless, our study provides novel contributions. The present study aimed to improve prediction by presenting overlapping evidence obtained through multi-bioinformatics rather than a single bioinformatics analysis. Despite the limitations described above, we were able to confirm the influence of the water extract on OXPHOS activity and ROS reduction. Further, it revealed that the two extracts can exhibit different activities and that COX5B is a major OXPHOS target of SF, representing a potential biomarker for drug efficacy.

## Figures and Tables

**Table 1:** Gene-specific primer pairs used for qPCR.

Targets	Forward (5′ → 3′)	Reverse (5′ → 3′)	Reference
*NDUFS5*	CGGTTATACTCGGGCAGAGAAAG	CTTATCCCGCTGCTTCCTGATG	NM_004552
*NDUFS7*	AGGCACGAGGTGTCCATCAGAG	CAGTTGACGAGGTCATCCAGCT	NM_024407
*COX5B*	GGAGATCATGCTGGCTGCAAAG	GCAGCCTACTATTCTCTTGTTGG	NM_001862
*GAPDH*	GTCTCCTCTGACTTCAACAGCG	ACCACCCTGTTGCTGTAGCCAA	NM_002046

**Table 2:** DTI network information based on docking according to four hydrophilicity groups.

DTI Network	Number of Nodes	Number of Compound Nodes	Number of Protein Nodes	Number of Edges
Strong hydrophilic DTI network	170	39	131	1476
Weak hydrophilic DTI network	183	72	111	1569
Weak hydrophobic DTI network	194	92	102	1436
Strong hydrophobic DTI network	201	69	132	1383

DTI, Drug–target interaction.

**Table 3:** PP DTI network information according to four hydrophilicity groups.

DTI Network	Number of Nodes	Number of Compound Nodes	Number of Protein Nodes	Number of Edges
Strong hydrophilic DTI network	1547	88	1459	4175
Weak hydrophilic DTI network	855	105	750	6582
Weak hydrophobic DTI network	1108	103	1005	7565
Strong hydrophobic DTI network	753	82	671	4995

PP, Polypharmacology; DTI, Drug–target interaction.

**Table 4:** Merged DTI network information based on docking and PP.

DTI Network	Number of Nodes	Number of Compound Nodes	Number of Protein Nodes	Number of Edges
Strong hydrophilic DTI network	83	45	38	451
Weak hydrophilic DTI network	120	92	28	437
Weak hydrophobic DTI network	122	94	28	344
Strong hydrophobic DTI network	111	78	33	378

DTI, Drug–target interaction; PP, Polypharmacology.

## Data Availability

The original contributions presented in this study are included in the article/Supplementary Materials. Further inquiries can be directed to the corresponding author. RNA-seq raw data for WESF and EESF are available in the GEO database (GSE294592, https://www.ncbi.nlm.nih.gov/geo/query/acc.cgi?acc=GSE294592, accessed on 5 May 2026).

## Supplementary Materials

The following supporting information can be downloaded at: https://www.mdpi.com/article/10.3390/antiox15060728/s1, Figure S1: Large-scale molecular docking study results based on OXPHOS-related proteins; Figure S2: Differential effects of SF extracts on candidate gene expression in SW1783; Figure S3: Docking–PP integrated network analysis results; Table S1: DEG_SF_water_high dose; Table S2: DEG_SF_ethanol_high dose; Table S3: Oxidative phosphorylation_protein_information; Table S4: Schisandrae Fructus_compound_PubChem_information; Table S5: OXPHOS PPI network; Table S6: OXPHOS PPI network_louvain group; Table S7: Uniprot_Respiratory complex protein; Table S8: docking ratio score; Tables S9: docking_DTI_degree; Table S10: docking_DTI_NeighborhoodConnectivity; Table S11: Merged and firstshell DTI network information based on docking and polypharmacology; Material S1: Schisandrae Fructus_compound_pdbqt; Material S2: Schisandrae Fructus_protein_pdbqt; Material S3: Schisandrae Fructus_docking_result; Material S4: docking_DTI_network; Material S5: polyphamacology_valid_interaction; Material S6: polyphamacology_DTI_network; Material S7: docking_PP_merge_network; Material S8: docking_PP_merge_network_centrality; Material S9: docking_PP_merge_firtshell_network; Material S10: docking_PP_merge_firtshell_network_centrality.

## Abbreviations

The following abbreviations are used in this manuscript:

SF	Schisandrae Fructus
TAM	traditional Asian medicine
OXPHOS	oxidative phosphorylation
ROS	reactive oxygen species
WESF	water extracts of SF
EESF	ethanol extracts of SF
PP	polypharmacology
PPI	protein–protein interaction
DTI	drug–target interaction
DMSO	dimethyl sulfoxide
PBS	phosphate-buffered saline
ATCC	American Type Culture Collection
SCAR	sequence-characterized amplified region
RNA-seq	RNA-sequencing
DEG	differentially expressed gene
GSEA	gene set enrichment analysis
KEGG	Kyoto Encyclopedia of Genes and Genomes
KDE	Kernel Density Estimation
GO	gene ontology
SD	standard deviation
Spho	strong hydrophobic group
Wpho	weak hydrophobic group
Wphi	weak hydrophilic group
Sphi	strong hydrophilic group

## References

[1] Panossian A., Wikman G. (2008). Pharmacology of *Schisandra chinensis* Bail.: An overview of Russian research and uses in medicine. J. Ethnopharmacol..

[2] Kopustinskiene D.M., Bernatoniene J. (2021). Antioxidant Effects of Schisandra chinensis Fruits and Their Active Constituents. Antioxidants.

[3] Guo M., An F., Wei X., Hong M., Lu Y. (2017). Comparative Effects of Schisandrin A, B, and C on Acne-Related Inflammation. Inflammation.

[4] Sowndhararajan K., Deepa P., Kim M., Park S.J., Kim S. (2018). An overview of neuroprotective and cognitive enhancement properties of lignans from Schisandra chinensis. Biomed. Pharmacother..

[5] Addissouky T.A., El Sayed I.E.T., Ali M.M., Alubiady M.H.S., Wang Y. (2024). Schisandra chinensis in liver disease: Exploring the mechanisms and therapeutic promise of an ancient Chinese botanical. Arch. Pharmacol. Ther..

[6] Zagorska-Dziok M., Wojciak M., Ziemlewska A., Niziol-Lukaszewska Z., Hoian U., Klimczak K., Szczepanek D., Sowa I. (2022). Evaluation of the Antioxidant, Cytoprotective and Antityrosinase Effects of Schisandra chinensis Extracts and Their Applicability in Skin Care Product. Molecules.

[7] Qu Y., Chan J.Y., Wong C.W., Cheng L., Xu C., Leung A.W., Lau C.B. (2015). Antidiabetic Effect of Schisandrae Chinensis Fructus Involves Inhibition of the Sodium Glucose Cotransporter. Drug Dev. Res..

[8] Jin D., Zhao T., Feng W.W., Mao G.H., Zou Y., Wang W., Li Q., Chen Y., Wang X.T., Yang L.Q. (2016). Schisandra polysaccharide increased glucose consumption by up-regulating the expression of GLUT-4. Int. J. Biol. Macromol..

[9] Park H.J., Cho J.Y., Kim M.K., Koh P.O., Cho K.W., Kim C.H., Lee K.S., Chung B.Y., Kim G.S., Cho J.H. (2012). Anti-obesity effect of in 3T3-L1 cells and high fat diet-induced obese rats. Food Chem..

[10] Lam P.Y., Yan C.W., Chiu P.Y., Leung H.Y., Ko K.M. (2011). Schisandrin B protects against solar irradiation-induced oxidative stress in rat skin tissue. Fitoterapia.

[11] Kim Y.J., Yoo S.R., Chae C.K., Jung U.J., Choi M.S. (2014). Omija fruit extract improves endurance and energy metabolism by upregulating PGC-1alpha expression in the skeletal muscle of exercised rats. J. Med. Food.

[12] Kim J.W., Ku S.K., Han M.H., Kim K.Y., Kim S.G., Kim G.Y., Hwang H.J., Kim B.W., Kim C.M., Choi Y.H. (2015). The administration of Fructus Schisandrae attenuates dexamethasone-induced muscle atrophy in mice. Int. J. Mol. Med..

[13] Chen W.W., Zhang X., Huang W.J. (2016). Role of neuroinflammation in neurodegenerative diseases (Review). Mol. Med. Rep..

[14] Zhao X., Liu C., Xu M., Li X., Bi K., Jia Y. (2016). Total Lignans of Schisandra chinensis Ameliorates Abeta1-42-Induced Neurodegeneration with Cognitive Impairment in Mice and Primary Mouse Neuronal Cells. PLoS ONE.

[15] Li X., Fang P., Mai J., Choi E.T., Wang H., Yang X.F. (2013). Targeting mitochondrial reactive oxygen species as novel therapy for inflammatory diseases and cancers. J. Hematol. Oncol..

[16] Li X., Fang P., Yang W.Y., Chan K., Lavallee M., Xu K., Gao T., Wang H., Yang X. (2016). Endothelial mitochondrial ROS, un-coupled from ATP synthesis, determine both physiological endothelial activation for recruitment of patrolling cells, and pathological recruitment of inflammatory cells. Can. J. Physiol. Pharmacol..

[17] Dash U.C., Bhol N.K., Swain S.K., Samal R.R., Nayak P.K., Raina V., Panda S.K., Kerry R.G., Duttaroy A.K., Jena A.B. (2025). Oxidative stress and inflammation in the pathogenesis of neurological disorders: Mechanisms and implications. Acta Pharm. Sin. B.

[18] Chen Y., Qin C., Huang J., Tang X., Liu C., Huang K., Xu J., Guo G., Tong A., Zhou L. (2020). The role of astrocytes in oxidative stress of central nervous system: A mixed blessing. Cell Prolif..

[19] Yan T., Shang L., Wang M., Zhang C., Zhao X., Bi K., Jia Y. (2016). Lignans from Schisandra chinensis ameliorate cognition deficits and attenuate brain oxidative damage induced by D-galactose in rats. Metab. Brain Dis..

[20] Ruisi Z. (2024). Introduction of Western Medicines into China and Improvement on Decoctions during Modern China. Chin. Med. Cult..

[21] Yang J.Y., Kim G.R., Chae J.S., Kan H., Kim S.S., Hwang K.S., Lee B.H., Yu S., Moon S., Park B. (2019). Antioxidant and anti-inflammatory effects of an ethanol fraction from the Schisandra chinensis baillon hot water extract fermented using Lactobacilius paracasei subsp. tolerans. Food Sci. Biotechnol..

[22] Bae J.Y., Lee Y.S., Han S.Y., Jeong E.J., Lee M.K., Kong J.Y., Lee D.H., Cho K.J., Lee H.S., Ahn M.J. (2012). A Comparison between Water and Ethanol Extracts of Rumex acetosa for Protective Effects on Gastric Ulcers in Mice. Biomol. Ther..

[23] Cieniak C., Walshe-Roussel B., Liu R., Muhammad A., Saleem A., Haddad P.S., Cuerrier A., Foster B.C., Arnason J.T. (2015). Phytochemical Comparison of the Water and Ethanol Leaf Extracts of the Cree medicinal plant, *Sarracenia purpurea* L. (Sarraceniaceae). J. Pharm. Pharm. Sci..

[24] Xiang J.Y., Chi Y.Y., Han J.X., Shi X., Cai Y., Xiang H., Xie Q. (2022). Intestinal Microbiota Contributes to the Improvement of Alcoholic Hepatitis in Mice Treated with Schisandra chinensis Extract. Front. Nutr..

[25] Zhang Y., Lv X., Qu J., Zhang X., Zhang M., Gao H., Zhang Q., Liu R., Xu H., Li Q. (2020). A systematic strategy for screening therapeutic constituents of *Schisandra chinensis* (Turcz.) Baill infiltrated blood-brain barrier oriented in lesions using ethanol and water extracts: A novel perspective for exploring chemical material basis of herb medicines. Acta Pharm. Sin. B.

[26] Park M., Baek S.J., Park S.M., Yi J.M., Cha S. (2023). Comparative study of the mechanism of natural compounds with similar structures using docking and transcriptome data for improving in silico herbal medicine experimentations. Brief. Bioinform..

[27] Fang J., Liu C., Wang Q., Lin P., Cheng F. (2018). In silico polypharmacology of natural products. Brief. Bioinform..

[28] Chaudhari R., Tan Z., Huang B., Zhang S. (2017). Computational polypharmacology: A new paradigm for drug discovery. Expert Opin. Drug Discov..

[29] Baek S.J., Lee H., Park S.M., Park M., Yi J.M., Kim N.S., Kim A., Cha S. (2022). Identification of a novel anticancer mechanism of Paeoniae Radix extracts based on systematic transcriptome analysis. Biomed. Pharmacother..

[30] Park S.M., Kim A., Lee H., Baek S.J., Kim N.S., Park M., Yi J.M., Cha S. (2022). Systematic transcriptome analysis reveals molecular mechanisms and indications of bupleuri radix. Front. Pharmacol..

[31] Kim A., Kim Y.R., Park S.M., Lee H., Park M., Yi J.M., Cha S., Kim N.S. (2024). Jakyak-gamcho-tang, a decoction of Paeoniae Radix and Glycyrrhizae Radix et Rhizoma, ameliorates dexamethasone-induced muscle atrophy and muscle dysfunction. Phytomedicine.

[32] Barabasi A.L., Oltvai Z.N. (2004). Network biology: Understanding the cell’s functional organization. Nat. Rev. Genet..

[33] Hopkins A.L. (2008). Network pharmacology: The next paradigm in drug discovery. Nat. Chem. Biol..

[34] Park M., Yi J.M., Kim N.S., Lee S.Y., Lee H. (2024). Effect of Poria cocos Terpenes: Verifying Modes of Action Using Molecular Docking, Drug-Induced Transcriptomes, and Diffusion Network Analyses. Int. J. Mol. Sci..

[35] Andrews S. FastQC: A Quality Control Tool for High Throughput Sequence Data. https://www.bioinformatics.babraham.ac.uk/projects/fastqc/.

[36] F. K. Trim Galore: A Wrapper Tool Around Cutadapt and FastQC to Consistently Apply Quality and Adapter Trimming to FastQ Files. https://www.bioinformatics.babraham.ac.uk/projects/trim_galore/.

[37] Dobin A., Davis C.A., Schlesinger F., Drenkow J., Zaleski C., Jha S., Batut P., Chaisson M., Gingeras T.R. (2013). STAR: Ultrafast universal RNA-seq aligner. Bioinformatics.

[38] Li B., Dewey C.N. (2011). RSEM: Accurate transcript quantification from RNA-Seq data with or without a reference genome. BMC Bioinform..

[39] Love M.I., Huber W., Anders S. (2014). Moderated estimation of fold change and dispersion for RNA-seq data with DESeq2. Genome Biol..

[40] Subramanian A., Tamayo P., Mootha V.K., Mukherjee S., Ebert B.L., Gillette M.A., Paulovich A., Pomeroy S.L., Golub T.R., Lander E.S. (2005). Gene set enrichment analysis: A knowledge-based approach for interpreting genome-wide expression profiles. Proc. Natl. Acad. Sci. USA.

[41] Liberzon A., Birger C., Thorvaldsdottir H., Ghandi M., Mesirov J.P., Tamayo P. (2015). The Molecular Signatures Database (MSigDB) hallmark gene set collection. Cell Syst..

[42] Korotkevich G., Sukhov V., Budin N., Shpak B., Artyomov M.N., Sergushichev A. (2016). Fast gene set enrichment analysis. bioRxiv.

[43] Benjamini Y., Hochberg Y. (1995). Controlling the false discovery rate: A practical and powerful approach to multiple testing. J. R. Stat. Soc. Ser. B Methodol..

[44] Kanehisa M., Furumichi M., Tanabe M., Sato Y., Morishima K. (2017). KEGG: New perspectives on genomes, pathways, diseases and drugs. Nucleic Acids Res..

[45] Safran M., Dalah I., Alexander J., Rosen N., Iny Stein T., Shmoish M., Nativ N., Bahir I., Doniger T., Krug H. (2010). GeneCards Version 3: The human gene integrator. Database.

[46] Kuleshov M.V., Jones M.R., Rouillard A.D., Fernandez N.F., Duan Q., Wang Z., Koplev S., Jenkins S.L., Jagodnik K.M., Lachmann A. (2016). Enrichr: A comprehensive gene set enrichment analysis web server 2016 update. Nucleic Acids Res..

[47] Maglott D., Ostell J., Pruitt K.D., Tatusova T. (2011). Entrez Gene: Gene-centered information at NCBI. Nucleic Acids Res..

[48] Ru J., Li P., Wang J., Zhou W., Li B., Huang C., Li P., Guo Z., Tao W., Yang Y. (2014). TCMSP: A database of systems pharmacology for drug discovery from herbal medicines. J. Cheminform..

[49] Liu Z., Guo F., Wang Y., Li C., Zhang X., Li H., Diao L., Gu J., Wang W., Li D. (2016). BATMAN-TCM: A Bioinformatics Analysis Tool for Molecular mechANism of Traditional Chinese Medicine. Sci. Rep..

[50] Kim S.K., Lee M.K., Jang H., Lee J.J., Lee S., Jang Y., Jang H., Kim A. (2024). TM-MC 2.0: An enhanced chemical database of medicinal materials in Northeast Asian traditional medicine. BMC Complement. Med. Ther..

[51] Bolton E.E., Chen J., Kim S., Han L., He S., Shi W., Simonyan V., Sun Y., Thiessen P.A., Wang J. (2011). PubChem3D: A new resource for scientists. J. Cheminform..

[52] Cheng T., Zhao Y., Li X., Lin F., Xu Y., Zhang X., Li Y., Wang R., Lai L. (2007). Computation of octanol-water partition coefficients by guiding an additive model with knowledge. J. Chem. Inf. Model..

[53] Miller M.M., Wasik S.P., Huang G.L., Shiu W.Y., Mackay D. (1985). Relationships between octanol-water partition coefficient and aqueous solubility. Environ. Sci. Technol..

[54] Jacobusse G., Veenman C. (2016). On selection bias with imbalanced classes. Proceedings of the International Conference on Discovery Science, Bari, Italy, 19–21 October 2016.

[55] Jumper J., Evans R., Pritzel A., Green T., Figurnov M., Ronneberger O., Tunyasuvunakool K., Bates R., Zidek A., Potapenko A. (2021). Highly accurate protein structure prediction with AlphaFold. Nature.

[56] O’Boyle N.M., Banck M., James C.A., Morley C., Vandermeersch T., Hutchison G.R. (2011). Open Babel: An open chemical toolbox. J. Cheminform..

[57] Trott O., Olson A.J. (2010). AutoDock Vina: Improving the speed and accuracy of docking with a new scoring function, efficient optimization, and multithreading. J. Comput. Chem..

[58] Szklarczyk D., Nastou K., Koutrouli M., Kirsch R., Mehryary F., Hachilif R., Hu D., Peluso M.E., Huang Q., Fang T. (2025). The STRING database in 2025: Protein networks with directionality of regulation. Nucleic Acids Res..

[59] Blondel V.D., Guillaume J.L., Lambiotte R., Lefebvre E. (2008). Fast unfolding of communities in large networks. J. Stat. Mech. Theory Exp..

[60] Hagberg A., Swart P.J., Schult D.A. Exploring network structure, dynamics, and function using NetworkX. Proceedings of the Python in Science Conference.

[61] Shannon P., Markiel A., Ozier O., Baliga N.S., Wang J.T., Ramage D., Amin N., Schwikowski B., Ideker T. (2003). Cytoscape: A software environment for integrated models of biomolecular interaction networks. Genome Res..

[62] UniProt Consortium (2025). UniProt: The Universal Protein Knowledgebase in 2025. Nucleic Acids Res..

[63] Maleki F., Ovens K., Hogan D.J., Kusalik A.J. (2020). Gene Set Analysis: Challenges, Opportunities, and Future Research. Front. Genet..

[64] Dogrusoz U., Giral E., Cetintas A., Civril A., Demir E. (2009). A layout algorithm for undirected compound graphs. Inf. Sci..

[65] Saxena A., Iyengar S. (2020). Centrality measures in complex networks: A survey. arXiv.

[66] Ali P.J.M., Faraj R.H., Koya E., Ali P.J.M., Faraj R.H. (2014). Data normalization and standardization: A technical report. Mach. Learn. Tech. Rep..

[67] Hearne A., Chen H., Monarchino A., Wiseman J.S. (2020). Oligomycin-induced proton uncoupling. Toxicol. Vitr..

[68] Gutenkunst R.N., Waterfall J.J., Casey F.P., Brown K.S., Myers C.R., Sethna J.P. (2007). Universally sloppy parameter sensitivities in systems biology models. PLoS Comput. Biol..

[69] Tiwari K., Kananathan S., Roberts M.G., Meyer J.P., Sharif Shohan M.U., Xavier A., Maire M., Zyoud A., Men J., Ng S. (2021). Reproducibility in systems biology modelling. Mol. Syst. Biol..

[70] Park M., Park S.M., Lee H., Kim A., Kim N.S., Kim Y.R., Yi J.M., Cha S. (2024). KORE-Map 1.0: Korean medicine Omics Resource Extension Map on transcriptome data of tonifying herbal medicine. Sci. Data.

[71] Chandrasekaran B., Abed S.N., Al-Attraqchi O., Kuche K., Tekade R.K. (2018). Computer-aided prediction of pharmacokinetic (ADMET) properties. Dosage form Design Parameters.

[72] Kamalov F., Sulieman H., Alzaatreh A., Emarly M., Chamlal H., Safaraliev M. (2025). Mathematical methods in feature selection: A review. Mathematics.

[73] Gleich D., Seshadhri C. (2011). Neighborhoods are good communities. arXiv.

[74] Evans T.S., Chen B.S. (2022). Linking the network centrality measures closeness and degree. Commun. Phys..

[75] Grossman L.I., Lomax M.I. (1997). Nuclear genes for cytochrome c oxidase. Biochim. Biophys. Acta.

[76] Uhlen M., Oksvold P., Fagerberg L., Lundberg E., Jonasson K., Forsberg M., Zwahlen M., Kampf C., Wester K., Hober S. (2010). Towards a knowledge-based Human Protein Atlas. Nat. Biotechnol..

[77] Chu Y.D., Lim S.N., Yeh C.T., Lin W.R. (2021). COX5B-Mediated Bioenergetic Alterations Modulate Cell Growth and Anticancer Drug Susceptibility by Orchestrating Claudin-2 Expression in Colorectal Cancers. Biomedicines.

[78] Gao S.P., Sun H.F., Jiang H.L., Li L.D., Hu X., Xu X.E., Jin W. (2015). Loss of COX5B inhibits proliferation and promotes senescence via mitochondrial dysfunction in breast cancer. Oncotarget.

[79] Gao S.P., Sun H.F., Fu W.Y., Li L.D., Zhao Y., Chen M.T., Jin W. (2017). High expression of COX5B is associated with poor prognosis in breast cancer. Futur. Oncol..

[80] Hu T., Xi J. (2017). Identification of COX5B as a novel biomarker in high-grade glioma patients. OncoTargets Ther..

[81] Chu Y.D., Lin W.R., Lin Y.H., Kuo W.H., Tseng C.J., Lim S.N., Huang Y.L., Huang S.C., Wu T.J., Lin K.H. (2020). COX5B-Mediated Bioenergetic Alteration Regulates Tumor Growth and Migration by Modulating AMPK-UHMK1-ERK Cascade in Hepatoma. Cancers.

[82] Pham L., Arroum T., Wan J., Pavelich L., Bell J., Morse P.T., Lee I., Grossman L.I., Sanderson T.H., Malek M.H. (2024). Regulation of mitochondrial oxidative phosphorylation through tight control of cytochrome c oxidase in health and disease—Implications for ischemia/reperfusion injury, inflammatory diseases, diabetes, and cancer. Redox Biol..

[83] Zhao Y., Sun X., Nie X., Sun L., Tang T.S., Chen D., Sun Q. (2012). COX5B regulates MAVS-mediated antiviral signaling through interaction with ATG5 and repressing ROS production. PLoS Pathog..

