# Supplementary material for: Multi-Bioinformatics Approach Reveals COX5B-Mediated Modulation of Oxidative Phosphorylation and ROS Levels by Water and Ethanol Extracts of Schisandrae Fructus in SW1783 Cells
Source: Antioxidants (Basel). 2026 Jun 9;15(6):728. doi: 10.3390/antiox15060728 (PMC13295596; doi:10.3390/antiox15060728)
Supplement: Supplementary file 1 [file antioxidants-15-00728-s001.zip › Supplementary Figures.pdf]

SUPPLEMENTARY MATERIALS

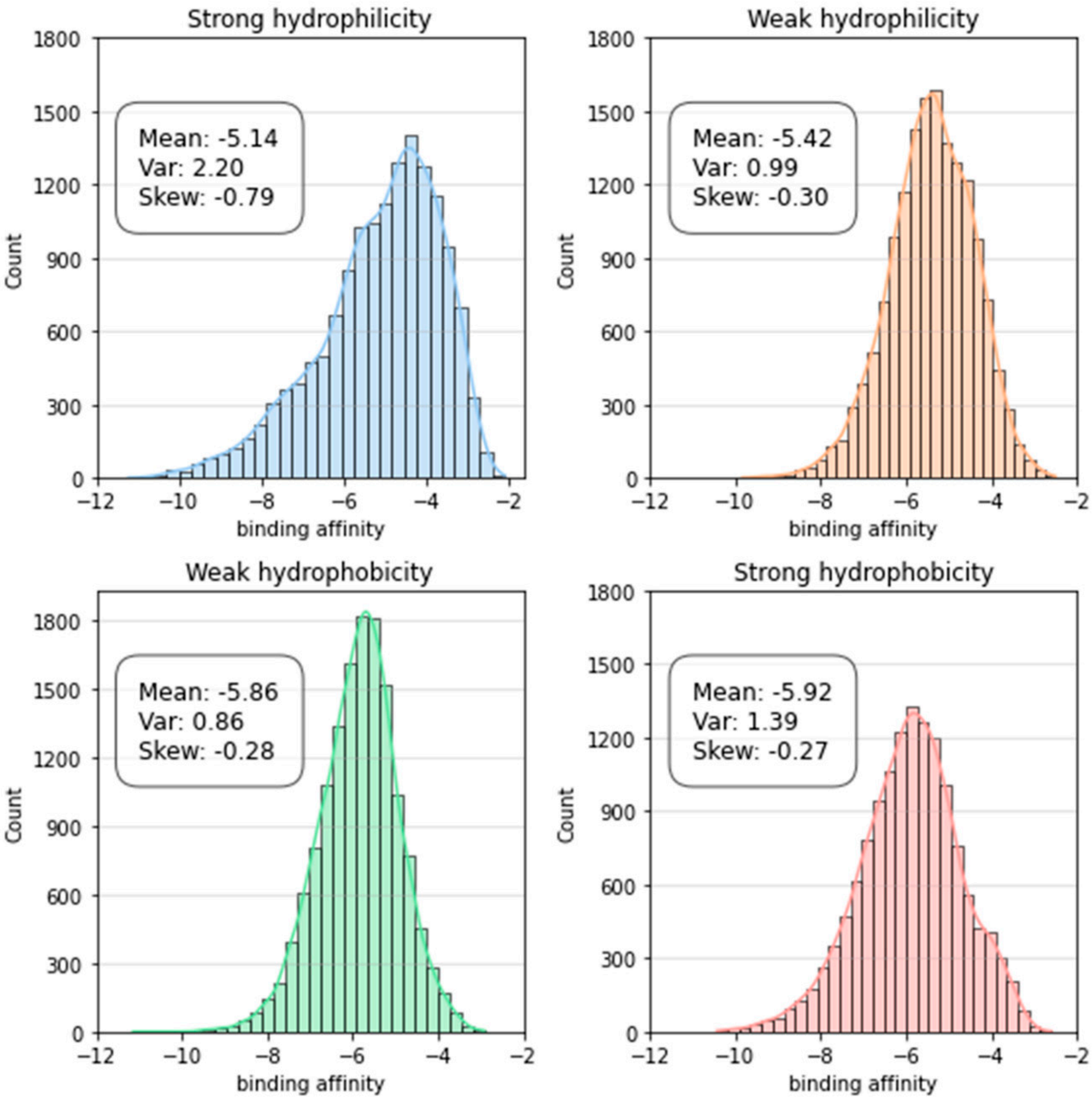

Supplementary Figure S1. Large-scale molecular docking study results based on OXPHOS-related proteins. The docking results for groups classified by hydrophilicity. The binding affinity values of the docking results for each group were visualized using histogram and kernel density estimation plot. The mean, variance, and skewness values for each group were calculated, and descriptive statistics are presented. OXPHOS, oxidative phosphorylation.

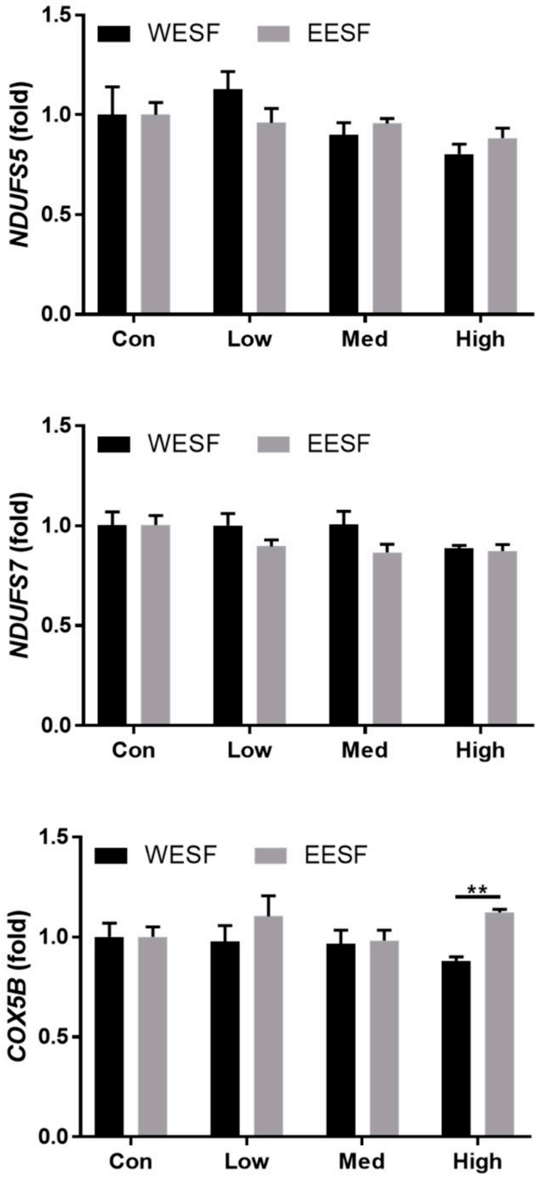

Supplementary Figure S2. Differential effects of SF extracts on candidate gene expression in SW1783. The effects of SF treatment on the expression of NDUFS5, NDUFS7, and COX5B were determined using qPCR analysis. SF, Schisandrae Fructus; WESF, hot water extract of SF; EESF, ethanol extract of SF. Low (20 µg/mL), medium (100 µg/mL), high (500 µg/mL) for WESF. Low (12.4 µg/mL), medium (62 µg/mL), high (310 µg/mL) for EESF. The data are presented as mean ± SD (n = 3).

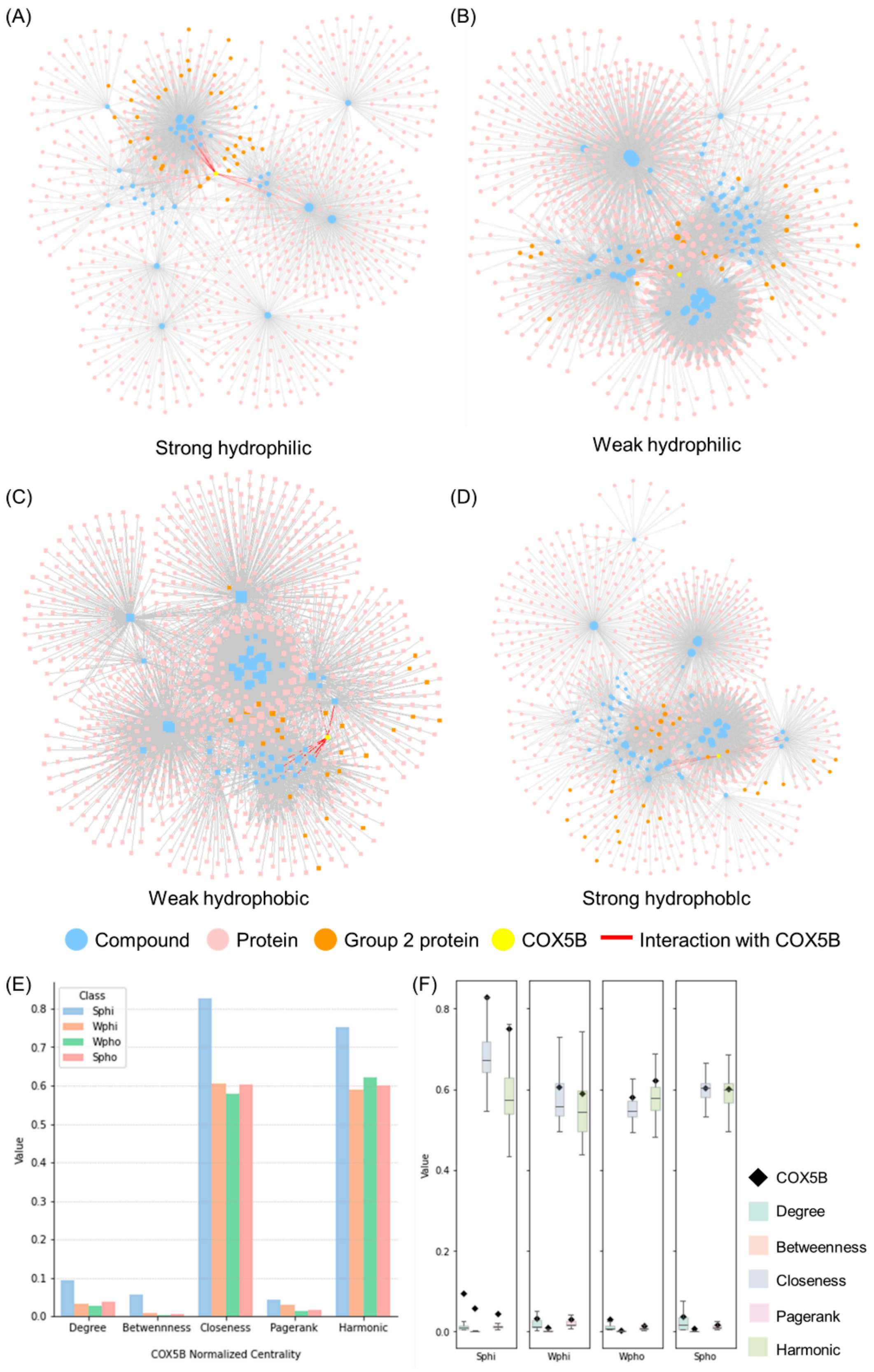
(A)
(B)
Strong hydrophilic
Weak hydrophilic
(C)
(D)
Weak hydrophobic
Strong hydrophoblc
Compound
Protein
Group 2 protein
COX5B
Interaction with COX5B
(E)
Class
Sphi
Wphi
Wpho
Spho
Value
0.0
0.1
0.2
0.3
0.4
0.5
0.6
0.7
0.8
Degree
Betwennness
Closeness
Pagerank
Harmonic
COX5B Normalized Centrality
(F)
Value
0.0
0.2
0.4
0.6
0.8
Sphi
Wphi
Wpho
Spho
COX5B
Degree
Betweenness
Closeness
Pagerank
Harmonic

Supplementary Figure S3. Docking–PP integrated network analysis results. (A–D) This network was reconstructed using the major proteins of the docking-based DTI network and the PP DTI network. The two networks were integrated for each group classified based on hydrophilicity, using the compounds linked to the main protein and proteins with two paths from the main protein. The blue nodes are SF compounds, the orange nodes are group 2 proteins selected from Figure 3F, which are major proteins, and the pink nodes are other proteins. The yellow node is COX5B, and the red line is a component predicted to interact with COX5B. (E) This is the result of calculating the centrality value of COX5B in the A-D network. (F) This is the result of calculating the relative centrality distribution of COX5B in the A-D network. The box plot shows the distribution of the centrality values of all nodes in the network, and the diamond-shaped dots indicate the location of COX5B. PP, polypharmacology; DTI, drug-target interaction.
